# Thermodynamics-inspired explanations of artificial intelligence

**DOI:** 10.1038/s41467-024-51970-x

**Published:** 2024-09-09

**Authors:** Shams Mehdi, Pratyush Tiwary

**Affiliations:** 1 Biophysics Program and Institute for Physical Science and Technology, University of Maryland, College Park, 20742 USA; 2 Department of Chemistry and Biochemistry and Institute for Physical Science and Technology, University of Maryland, College Park, 20742 USA; 3 University of Maryland Institute for Health Computing, Bethesda, Maryland 20852 USA

**Keywords:** Statistical mechanics, Method development, Cheminformatics, Information theory and computation

## Abstract

In recent years, predictive machine learning models have gained prominence across various scientific domains. However, their black-box nature necessitates establishing trust in them before accepting their predictions as accurate. One promising strategy involves employing explanation techniques that elucidate the rationale behind a model’s predictions in a way that humans can understand. However, assessing the degree of human interpretability of these explanations is a nontrivial challenge. In this work, we introduce interpretation entropy as a universal solution for evaluating the human interpretability of any linear model. Using this concept and drawing inspiration from classical thermodynamics, we present Thermodynamics-inspired Explainable Representations of AI and other black-box Paradigms, a method for generating optimally human-interpretable explanations in a model-agnostic manner. We demonstrate the wide-ranging applicability of this method by explaining predictions from various black-box model architectures across diverse domains, including molecular simulations, text, and image classification.

## Abstract (web summary)

Predictive machine learning models, while powerful, are often seen as black boxes. Here, the authors introduce a thermodynamics-inspired approach for generating rationale behind their explanations across diverse domains based on the proposed concept of interpretation entropy.

## Introduction

Performing predictions based on observed data is a general problem of interest in a wide range of scientific disciplines. Traditionally, scientists have tackled this problem by developing mathematical models that connect observations with predictions using their knowledge of the underlying physical processes. However, in many practical situations, constructing such explicit models is unfeasible due to a lack of system-specific information^1^. In recent years, an alternative class of purely data-driven approaches involving Artificial Intelligence (AI) has emerged with remarkable success^2–9^. These methods are often referred to as black-box models, as they don’t rely on a deep understanding of the system’s inner workings and are designed to extract patterns directly from data. However, when it comes to making informed decisions and policies based on these models, this lack of understanding raises concerns.

Recently there has been significant progress in addressing this issue and the proposed approaches can be classified into two categories: (1) AI models that are inherently explainable (e.g., decision trees providing understandable decision paths^10^, scoring mechanisms^11,12^, generalized additive models, etc.^13,14^), or (2) post-hoc explanation schemes for AI models that are not inherently explainable called XAI (e.g., gradient-based methods: layer-wise relevance propagation (LRP)^15^, guided back-propagation^16^, integrated gradients^17^; tree^18^, or linear^19^ surrogate models approximating black-box behavior; approaches based on game theory^20^, etc.). Although there has been a recent push toward the former class of methods due to certain limitations of XAI^21^, most of the existing black-box AI are not inherently explainable. Consequently, XAI has been widely adopted for generating human comprehensible rationale for black-box AI predictions^22^. Under the XAI paradigm, the developed methods can be black-box model-specific, or model-agnostic that generate global or locally valid explanations in the form of visual or feature importance attributions^23–25^.

In this work, we focus on model-agnostic XAI approaches, i.e., a specific class of methods that work by accessing only the input and output layers of a black-box model. Recently, there has been a trend where more and more ML models are being released only for inference purposes at the user level while the model architecture and trained parameters are reserved for commercial purposes. To assess the trustworthiness of such ML models, model-agnostic XAI is one of the few effective choices.

One of the earliest and most influential model-agnostic explanation methods is the Partial Dependence Plot (PDP)^26^. PDPs visualize the relationship between a subset of features and the prediction while holding all other features constant. Much later, in 2016, a significant breakthrough in model-agnostic explanations came with the introduction of Local Interpretable Model-agnostic Explanations (LIME) by Ribeiro et al.^19^ LIME constructs a linear surrogate model that locally approximates the behavior of a black-box model. Coefficients associated with each feature of the constructed linear model are then used to attribute local feature importance. Due to its ease of use, LIME has become one of the most widely adopted model-agnostic explanation methods. In a subsequent work in 2018, Ribeiro et al. introduced Anchors^27^, a method that aims to identify sufficient if-then conditions as explanations that preserve a prediction when the feature values are changed. Since then, other researchers have worked on extending the applicability of LIME, e.g., Zhang et al.^28^ investigated potential uncertainties that can arise in LIME due to the randomized neighborhood sampling procedure, incorrect similarity measurement, lack of robustness, etc., and proposed a set of tests for trusting the explanations themselves.

SHapley Additive exPlanations (SHAP)^20^, introduced by Lundberg and Lee in 2017, further advanced the field by integrating cooperative game theory concepts with model-agnostic explanation methods. SHAP values offer a comprehensive metric for feature importance by evaluating each feature’s contribution to the prediction by taking into account all the possible sets of feature combinations. A key advantage of SHAP is its ability to detect non-linear dependencies among features. Furthermore, SHAP is capable of providing both local and global explanations for black-box predictions.

Although these methods have been developed to rationalize AI predictions, there is a potential issue ensuring high human interpretability. The challenge is that there are no established methods that directly quantify the degree of human interpretability of the generated explanations. This is a major concern in assessing AI model trustworthiness but is often overlooked. For instance, when rationalization involves a high number of correlated features, achieving high human interpretability and, consequently, establishing trust can be challenging. Research progress in this direction so far includes methods that construct linear models to approximate AI models and take the number of model parameters as a proxy for human interpretability (similar to some established methods in other mathematical domains, e.g., in Akaike information criterion^29^ or Bayesian information criterion^30^).

**Fig. 1:**
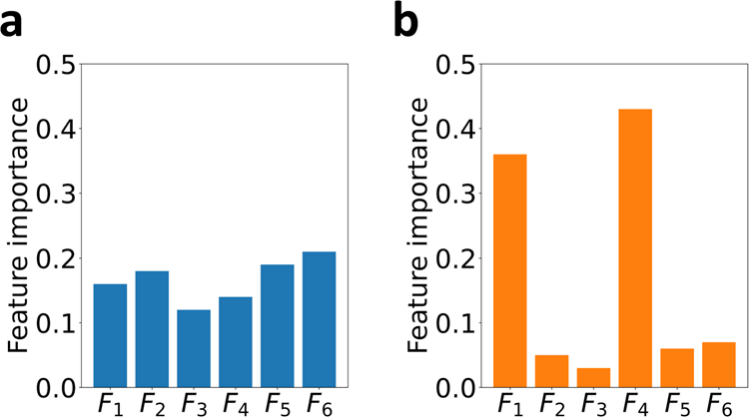
Model complexity is not a good descriptor for human interpretability. **a** Illustrative input feature coefficients for linear model 1. **b** Coefficients for linear model 2. Both models have the same number of model parameters (six). However, model 2 is significantly more human-interpretable than model 1, where two of the six features stand out as most relevant for predictions.

One of the primary motivations behind our work is the recognition that model complexity can be an insufficient descriptor of human interpretability, as shown in Fig. 1. In this case, if model complexity is used as a proxy for human interpretability, then both linear models shown in Fig. 1a, b will be assigned the same value as they both have the same number of model parameters. Indeed, previous studies^31–33^ have revealed constraints in human cognition arising from a bottleneck in information processing capacity when subjected to different stimuli. Thus, we ground ourselves in the information-theoretic definition of entropy^34^ and adopt a methodology that views linear model weights as a probability distribution. This allows us to assess differences in human interpretability among the different linear models by calculating a quantity similar to Shannon entropy. As illustrated in Fig. 1, it is evident that model 2 is significantly more understandable to humans compared to model 1. If both models exhibit equal accuracy, then a selection of model 2 over 1 is desirable, since it provides fewer actionable strategies. We solve this problem in the existing methods by introducing the concept of interpretation entropy for assessing the degree of human interpretability of any linear model. We show that under simple conditions, our definition of interpretation entropy addresses the shortcomings of complexity-based quantification.

Furthermore, we view the overall problem of AI model explanation from the lens of classical thermodynamics^35^. It is known in thermodynamics that the equilibrium state of a system is characterized by a minimum in its Helmholtz Free Energy *F*(*T*, *V*) ≔ *U* − *TS*. Here *U* and *S* represent the internal energy and entropy, respectively, of a system with a fixed number of particles *N* at constant temperature *T* and volume *V*. Similarly, we set up a formalism in this work where the optimality of an explanation (*ζ*) is assessed as a trade-off between its unfaithfulness (${{{\mathcal{U}}}}$) to the underlying ground truth, and interpretation entropy (${{{\mathcal{S}}}}$). Similar to *U* and *S* in classical thermodynamics, in our formalism ${{{\mathcal{U}}}}$ and ${{{\mathcal{S}}}}$ depend monotonically on each other. The strength of this trade-off can be tuned to identify the most stable explanation using a parameter *θ*, which plays a role similar to thermodynamic temperature *T*. For any choice of *θ* > 0, *ζ* is then guaranteed to have exactly one minimum characterized by a pair of values $\{{{{\mathcal{U}}}},{{{\mathcal{S}}}}\}$ under certain conditions.

We call our approach Thermodynamics-inspired Explainable Representations of AI and other black-box Paradigms (TERP), which takes inspiration from LIME and constructs local, linear surrogate models for generating black-box explanations. However, as opposed to the methods in existing literature, TERP focuses on directly quantifying the degree of human interpretability using the concept of interpretation entropy introduced in this work to generate a unique explanation. Owing to its model-agnostic implementation, TERP can be used for explaining predictions from any AI classifier. We demonstrate this generality by explaining predictions from the following black-box models in this work: (1) autoencoder-based VAMPnets^36^ for tabular molecular data, (2) self-attention-based vision transformers for images^37^ and, (3) attention-based bidirectional long short-term memory (Att-BLSTM) for text^38^ classification. In particular, the first class of models belongs to an area of research undergoing rapid progress involving molecular dynamics (MD) simulations^39–51^. As researchers with a keen interest in MD simulations, we have observed that the application of AI explanation tools to AI models in this field has been very limited. Consequently, we believe that our proposed method, TERP, will prove valuable to the broader scientific community focused on this subject.

## Results

### Interpretation unfaithfulness (${{{\mathcal{U}}}}$) for surrogate model construction

Our starting point is some given dataset ${{{\mathcal{X}}}}$ and corresponding predictions *g* coming from a black-box model. For a particular element $x\in {{{\mathcal{X}}}}$, we seek explanations that are as human-interpretable as possible while also being as faithful as possible to *g* in the vicinity of *x*. We aim to address this problem of explaining *g* by developing a linear approximation instead, which is more interpretable due to its linear construction. Specifically, we formulate *F* as a linear combination of an ordered set of representative features, *s* = {*s*_1_, *s*_2_, …, *s*_*n*_}. Typically, these features are domain-dependent, e.g., one-hot encoded superpixels for an image, keywords for text, and standardized values for tabular data. We demonstrate this in Equation (1) below, where *F* represents the linear approximation, *f*_0_ is a constant, and *f*_*k*_ comes from an ordered set of feature coefficients, *f* = {*f*_1_, *f*_2_, …, *f*_*n*_}.

1 $$F={f}_{\! 0}+{\Sigma }_{k=1}^{n} \, \, {f}_{k}{s}_{k}$$

Let’s consider a specific problem where **x**_**0**_ is a high-dimensional instance, and *g*(**x**_**0**_) is a black-box model prediction, for which an explanation is needed. We first generate a neighborhood {**x**_**1**_, **x**_**2**_, …, **x**_**N**_} of *N* samples by randomly perturbing the high-dimensional input space^22^. A detailed discussion of neighborhood generation is provided in “Methods.” Afterward, the black-box predictions {*g*(**x**_**1**_), *g*(**x**_**2**_), …, *g*(**x**_**N**_)} associated with each sample in the neighborhood are obtained. Subsequently, a local surrogate model is constructed by employing linear regression using the loss function defined in Equation (2).

2 $${{{\mathcal{L}}}}={\min }_{{f}_{k}}\mathop{\sum }_{i=1}^{N}{\Pi }_{i}({{{{\bf{x}}}}}_{{{{\bf{0}}}}},{{{{\bf{x}}}}}_{{{{\bf{i}}}}}){\left[g({{{{\bf{x}}}}}_{{{{\bf{i}}}}})-\left({\sum }_{k=1}^{n}{f}_{k}{s}_{ik}\right)\right]}^{2}$$

Here ${\Pi }_{i}({{{{\bf{x}}}}}_{{{{\bf{0}}}}},{{{{\bf{x}}}}}_{{{{\bf{i}}}}})={e}^{-d{({{{{\bf{x}}}}}_{{{{\bf{0}}}}},{{{{\bf{x}}}}}_{{{{\bf{i}}}}})}^{2}/{\sigma }^{2}}$ is a Gaussian similarity measure, where *d* is the distance between the explanation instance **x**_**0**_ and a neighborhood sample **x**_**i**_. In previous surrogate model construction approaches^19^, Euclidean distance in the continuous input feature space has been the typical choice for *d*. However, if the input space has several correlated or redundant features, a similarity measure based on Euclidean distance can be misleading^52,53^. TERP addresses this problem by computing a one-dimensional (1-d) projection of the neighborhood using linear discriminant analysis^54^ (LDA), which removes redundancy and produces more accurate similarity. Such a projection encourages the formation of two clusters in a 1-d space, corresponding to in-class and not in-class data points, respectively, by minimizing within-class variance and maximizing between-class distances. Since the projected space is one-dimensional, there is no need to tune the hyperparameter, *σ* in ${\Pi }_{i}({{{{\bf{x}}}}}_{{{{\bf{0}}}}},{{{{\bf{x}}}}}_{{{{\bf{i}}}}})={e}^{-d{({{{{\bf{x}}}}}_{{{{\bf{0}}}}},{{{{\bf{x}}}}}_{{{{\bf{i}}}}})}^{2}/{\sigma }^{2}}$ as might be necessary in established methods, and we can set *σ* = 1. We demonstrate the advantages of LDA-based similarity for practical problems by performing experiments in a subsequent subsection.

Next, we introduce a meaningful unfaithfulness measure (${{{\mathcal{U}}}}$) of the generated interpretation, computed from the correlation coefficient *C* between linear, surrogate model predictions (*F*) obtained using Equation (1) and black-box predictions (*g*). For any interpretation, *C*(*F*, *g*) ∈ [ − 1, + 1], and thus interpretation unfaithfulness is bounded, i.e., ${{{\mathcal{U}}}}\in [0,1]$

3 $${{{\mathcal{U}}}}=1-| C(F,g)|$$

Using these definitions, we implement a forward feature selection scheme^55,56^ by first constructing *n* linear models, each with *j* = 1 non-zero coefficients. We use Equation (3) to identify the feature responsible for the lowest ${{{{\mathcal{U}}}}}^{j=1}$. Here, the superscript *j* = 1 highlights that ${{{\mathcal{U}}}}$ was calculated for a model with *j* = 1 non-zero coefficients. We will follow this notation for other relevant quantities throughout this manuscript.

Afterward, the selected feature is propagated to identify the best set of two features resulting in the lowest ${{{{\mathcal{U}}}}}^{\, j=2}$, and the scheme is continued until ${{{{\mathcal{U}}}}}^{\, j=n}$ is computed. Since a model with *j* + 1 non-zero coefficients will be less or at best equally unfaithful as a model with *j* non-zero coefficients as defined in Equation (1), it can be observed that ${{{\mathcal{U}}}}$ monotonically decreases with *j*. The overall scheme generates *n* distinct interpretations as *j* goes from 1 to *n*.

### Interpretation entropy (${{{\mathcal{S}}}}$) for model selection

After identifying *n* interpretations, our goal is to determine the optimal interpretation from this family of models. At this point, we introduce the definition of interpretation entropy ${{{\mathcal{S}}}}$ for quantifying the degree of human interpretability of any linear model. Given a linear model with an ordered set of feature coefficients {*f*_1_, *f*_2_, …, *f*_*n*_} among which *j* are non-zero, we can define {*p*_1_, *p*_2_, …, *p*_*n*_}, where ${p}_{k}:=\frac{| \, {f}_{k}| }{{\sum }_{i=1}^{n}| \, {f}_{i}| }$. Interpretation entropy is then defined as:

4 $${{{{\mathcal{S}}}}}^{\, \, j}=-\mathop{\sum }_{k=1}^{n}{p}_{k}\log {p}_{k}| \{\log {p}_{k}=0\,\forall \,{p}_{k}=0\}$$

Here the superscript *j* indicates that ${{{\mathcal{S}}}}$ is calculated for a model with *j* non-zero coefficients. It is easy to see that *p*_*k*_ satisfies the properties of a probability distribution. Specifically, *p*_*k*_ ≥ 0 and ${\sum }_{k=1}^{n}{p}_{k}=1$.

Similar to the concept of self-information/surprisal in information theory, the negative logarithm of *p*_*k*_ from a fitted linear model can be defined as the self-interpretability penalty of that feature. Interpretation entropy is then computed as the expectation value of self-interpretability penalty of all the features, as shown in Equation (5). Using Jensen’s inequality, it can be shown that ${{{\mathcal{S}}}}$ has an upper limit of $\log n$ and we can normalize the definition so that ${{{\mathcal{S}}}}$ is bounded between [0, 1].

5 $${{{{\mathcal{S}}}}}^{\, \, j}=\frac{-1}{\log n}\mathop{\sum }_{k=1}^{n}{p}_{k}\log {p}_{k}=\frac{1}{\log n}{\mathbb{E}}[-\log p]$$

This functional form of interpretation entropy (${{{\mathcal{S}}}}$), i.e., interpretability penalty, encourages low values for a sharply peaked distribution of fitted weights, indicating high human interpretability and vice versa. Furthermore, if the features are independent, ${{{\mathcal{S}}}}$ has two interesting properties expressed in the theorems below. The corresponding proofs are provided in Supplementary Notes 1 and 2 of the Supplementary Information (SI).

#### Theorem 1

${{{{\mathcal{S}}}}}^{\, j}$ is a monotonically increasing function of the number of features (*j*).

#### Theorem 2

${{{\mathcal{S}}}}$ monotonically increases as ${{{\mathcal{U}}}}$ decreases (Supplementary Fig. S1).

### Free energy (*ζ*) for optimal explanation

**Fig. 2:**
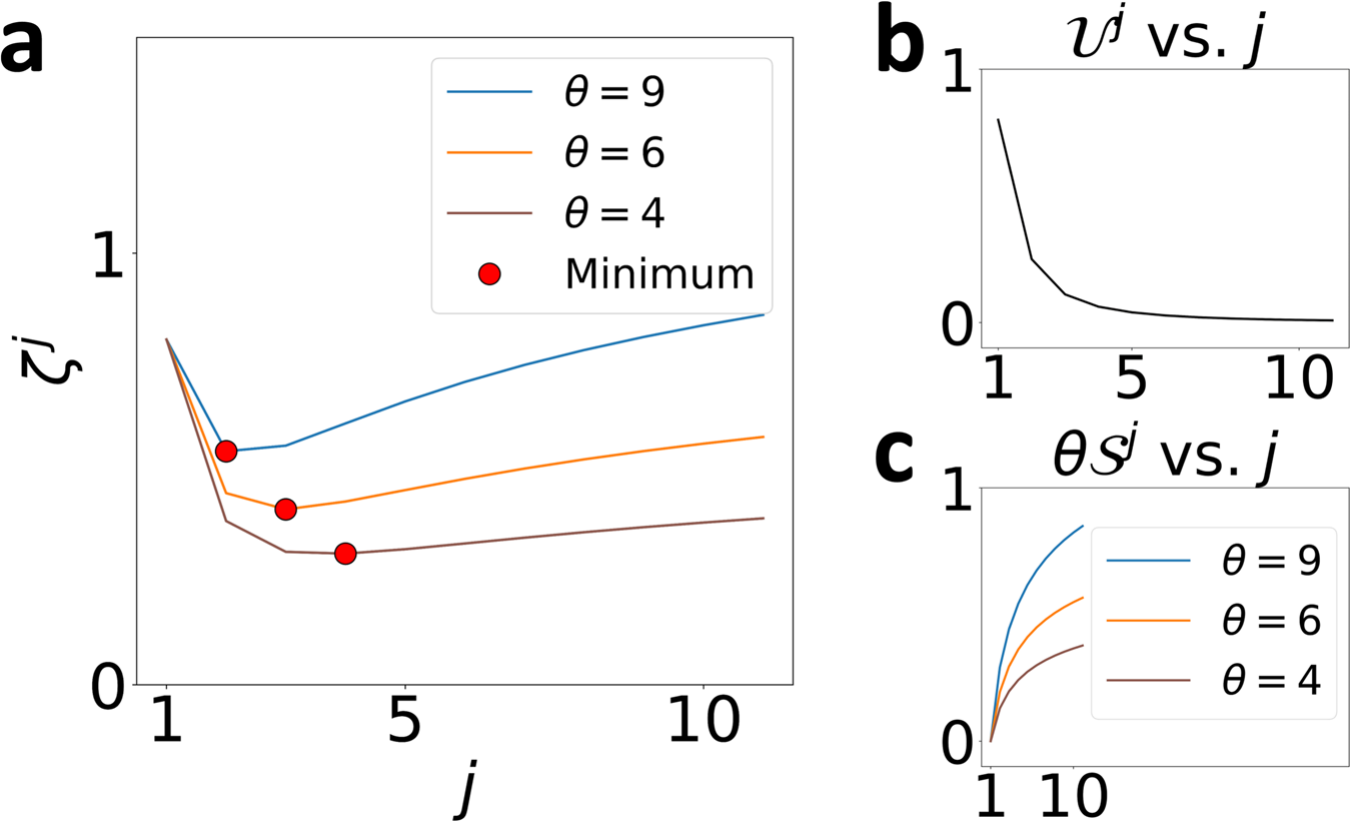
Illustrative example highlighting properties of free energy *ζ* ^*j*^, unfaithfulness ${{{{\mathcal{U}}}}}^{\, j}$, and interpretation entropy ${{{{\mathcal{S}}}}}^{\, j}$. **a** Strength of ${{{{\mathcal{S}}}}}^{\, j}$ contribution to *ζ* ^*j*^ can be tuned using *θ*. *ζ* ^*j*^ vs. *j* plots for three different *θ* = 9, 6, 4 are shown, resulting in minimums at *j* = 2, 3, 4, respectively. **b**${{{{\mathcal{U}}}}}^{\, \, j}$ vs. *j* remains unaffected by *θ*. **c**$\theta {{{{\mathcal{S}}}}}^{\, \, j}$ vs. *j* plot shows that the strength of the trade-off can be tuned by *θ*.

For an interpretation with *j* non-zero coefficients, we now define free energy *ζ* ^*j*^ as a trade-off between ${{{{\mathcal{U}}}}}^{\, j}$, and ${{{{\mathcal{S}}}}}^{\, \, j}$ tunable by a parameter *θ* ≥ 0, as shown in Fig. 2 and Equation (6).

6 $${\zeta }^{\, j}(\, \, f,\theta )={{{{\mathcal{U}}}}}^{\, \, j}+\theta {{{{\mathcal{S}}}}}^{\, \, j}$$

By writing an expression shown in Equation (7) for the stationary value, *Δζ* ^*j*^ = *ζ* ^*j*+1^ − *ζ* ^*j*^ = 0, we can define characteristic temperatures *θ* ^*j*^ at each *j* ∈ [1, *n* − 1]. Essentially, ${\theta }^{\, \, j}=-\frac{\Delta {{{{\mathcal{U}}}}}^{\, \, j}}{\Delta {{{{\mathcal{S}}}}}^{\, \, j}}$ is a measure of change in unfaithfulness per unit change in interpretation entropy for a model with *j* non-zero coefficients. This closely resembles the definition of thermodynamic temperature which is defined as the derivative of internal energy with respect to entropy. Afterward, we identify the interpretation with (*j* + 1) non-zero coefficients that minimizes $({\theta }^{\, \, j+1}-{\theta }^{\, \, j})=-(\frac{\Delta {{{{\mathcal{U}}}}}^{\, \, j+1}}{\Delta {{{{\mathcal{S}}}}}^{\, \, j+1}}-\frac{\Delta {{{{\mathcal{U}}}}}^{\, j}}{\Delta {{{{\mathcal{S}}}}}^{\, \, j}})$ as the optimal interpretation since it is guaranteed that *ζ* ^*j*+1^ will preserve the lowest minimum among the set {*ζ* ^1^, *ζ* ^2^, …, *ζ* ^*j*^, …, *ζ* ^*n*^} within the widest range of temperatures. Finally, we calculate optimal temperature, ${\theta }^{o}=\frac{{\theta }^{\, \, j+1}+{\theta }^{\, \, j}}{2}$ (any value within *θ* ^*j*^ < *θ* < *θ* ^*j*+1^ is equally valid since the optimal interpretation itself does not change) and generate the explanation as weights of this model. All *ζ* ^*j*^ vs. *j* plots shown in this manuscript are created using this definition of optimal temperature.

7 $$\begin{array}{rcl}{\zeta }^{j+1}-{\zeta }^{\, j}&=&({{{{\mathcal{U}}}}}^{j+1}-{{{{\mathcal{U}}}}}^{\, j})+\theta ({{{{\mathcal{S}}}}}^{j+1}-{{{{\mathcal{S}}}}}^{\, j})\\ \Delta {\zeta }^{\, j}&=&\Delta {{{{\mathcal{U}}}}}^{\, j}+\theta \Delta {{{{\mathcal{S}}}}}^{\, j}\\ {\theta }^{\, \, j}&=&-\frac{\Delta {{{{\mathcal{U}}}}}^{\, j}}{\Delta {{{{\mathcal{S}}}}}^{\, j}}[\,{\mbox{By setting}}\,\Delta {\zeta }^{\, j}=0]\end{array}$$

Thus,

8 $${\zeta }^{\, j}={{{{\mathcal{U}}}}}^{\, j}+\left(-\frac{\Delta {{{{\mathcal{U}}}}}^{\, j}}{\Delta {{{{\mathcal{S}}}}}^{\, j}}{| }_{\Delta {\zeta }^{\, j}=0}\right){{{{\mathcal{S}}}}}^{\, j}$$

**Fig. 3:**
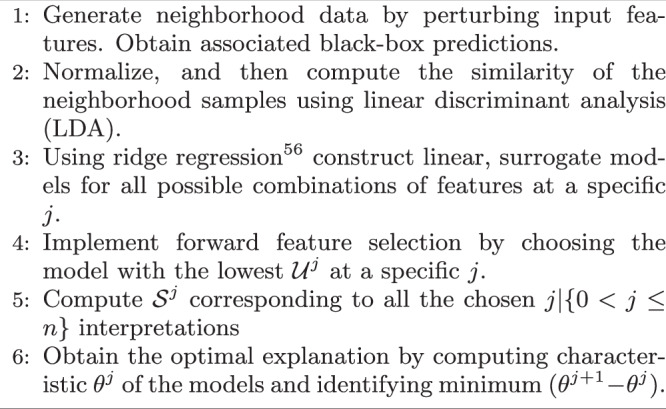
TERP algorithm. Describes the protocol to generate the optimal TERP explanation corresponding to a black-box model prediction.

This is again reminiscent of classical thermodynamics, where a system’s equilibrium configuration will, in general, vary with temperature, but the coarse-grained metastable state description remains robust over a well-defined range of temperatures (Supplementary Note 3). In our framework, when *θ* = 0, *ζ* ^*j*^ is minimized at *j* = *n* interpretation or the model that maximizes unfaithfulness and completely ignores entropy. As *θ* is increased from zero, interpretation entropy contributes more to *ζ* ^*j*^. Here, (*θ* ^*j*+1^ − *θ* ^*j*^ ) is a measure of the stability of the *j* non-zero coefficient interpretation. The complete TERP protocol is summarized as an algorithm in Fig. 3.

### Application to AI-augmented MD: VAMPnets

Variational approach for Markov processes (VAMPnets) is a popular technique for analyzing molecular dynamics (MD) trajectories^36^. VAMPnets can be used to featurize, transform inputs to a lower-dimensional representation, and construct a Markov state model^57^ in an automated manner by maximizing the so-called VAMP score. Additional details involving the implementation of VAMPnets are provided in “Methods.”

**Fig. 4:**
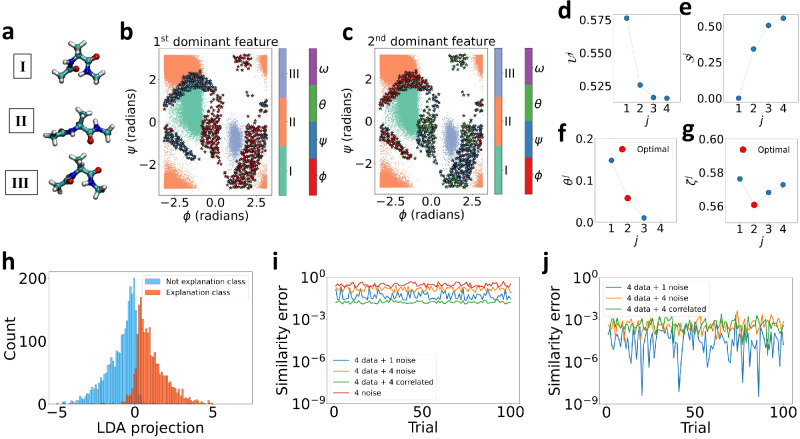
Using TERP to explain VAMPnets for molecular dynamics simulations of alanine dipeptide in vacuum. **a** Representative conformational states of alanine dipeptide labeled I, II, III. **b**, **c** Projected converged states are highlighted in three different colors as obtained by VAMPnets along (*ϕ*, *ψ*) dihedral angles. 713 different configurations are chosen for TERP. The first and second dominant features are highlighted using colored (⋆) in (**b**) and (**c**), respectively. **d**${{{{\mathcal{U}}}}}^{\, j}$ vs. *j*, **e**${{{{\mathcal{S}}}}}^{\, j}$ vs. *j*, **f***θ* ^*j*^ vs. *j*, and **g***ζ* ^*j*^ vs. *j* plots for a specific black-box prediction with configuration *ϕ* = 0.084, *ψ* = 0.007, *θ* = 0.237, *ω* = 2.990 radians, showing optimal interpretation occurring at *j* = 2. **h** High-dimensional neighborhood data projected onto 1-d using LDA for improved similarity measure. Binarizing the class prediction probabilities of the neighborhood using a threshold of 0.5 results in explanation and not explanation classes, respectively. The LDA projection separates the two regimes of prediction probability, showing meaningful projection. Average similarity error, *ΔΠ* defined in Equation (9) per datapoint for **i** Euclidean, and **j** LDA-based similarity, respectively. Comparison between (**i**) and (**j**) shows minimal error for LDA-based similarity, specifically demonstrated for an input space constructed from the four dihedral angles plus one pure noise, four pure noise, and four correlated features with partial noise, respectively. The input space for no actual data and four pure noise features in (**i**) establishes a baseline, showing that the Euclidean similarity will include significant error even when one redundant feature is included. All the calculations were performed in 100 independent trials to appropriately examine the effects.

In this work, we trained a VAMPnets model on a standard toy system: alanine dipeptide in vacuum. An 8-dimensional input space with sines and cosines of all the dihedral angles *ϕ*, *ψ*, *θ*, *ω* was constructed and passed to VAMPnets. VAMPnets was able to identify three metastable states I, II, and III as shown in Fig. 4b, c.

To explain VAMPnets model predictions using TERP, we picked 713 different configurations, some of which are near different transition states. To quantify data points as being a transition state, we use the criterion that the prediction probability for both classes should be higher than a threshold of 0.4. From a physics perspective, the behavior of such molecular systems near the transition states is a very pertinent question. Additionally, class prediction probability is the most sensitive at the transition state, and if our method generates a meaningful local neighborhood, it should include a broad distribution of probabilities resulting in highly accurate approximations to the black-box behavior. Thus, a correct analysis of the transition state ensemble will validate our similarity metric and overall neighborhood generation scheme.

We generated 5000 neighborhood samples for each configuration and performed TERP by following the algorithm in Fig. 3. In Fig. 4b, c, we highlight the first, and second most dominant features using colored stars (⋆) identified by TERP for all the 713 configurations. The generated explanations are robust and TERP identified various regions where different dihedral angles are relevant to predictions. The results are in agreement with existing literature, e.g., the relevance of *θ* dihedral angle at the transition state between I and III as reported by Chandler et al.^58^. Also, the results intuitively make sense, e.g., we see the VAMPnets state definitions change rapidly near *ϕ* ≈ 0, and TERP learned that *ϕ* is the most dominant feature in that region. This shows that VAMPnets worked here for the correct reasons and can be trusted. In Fig. 4d–g, we show TERP results for a specific configuration (*ϕ* = 0.084, *ψ* = 0.007, *θ* = 0.237, *ω* = 2.990 radians) for which *j* = 2 non-zero model resulted in optimal interpretation with *p*_*ϕ*_ = 0.82, and *p*_*θ*_ = 0.18. Figure 4f clearly shows that (*θ* ^*j*+1^ − *θ* ^*j*^) is minimized at *j* = 2 and the average of *θ* ^*j*+1^, and *θj* is taken as the optimal temperature *θ* ^*o*^ for calculating *ζ* ^*j*^ using Equation (8). Additional implementation details are provided in “Methods.”

In this section, we demonstrated the applicability of TERP for probing black-box models designed to analyze time-series data coming from MD simulations. In addition to assigning confidence to these models, TERP can be used to extract valuable insights (relevant degrees of freedom) learned by the model. In the future, we expect an increased adoption of TERP-like methods in the domain of AI-enhanced MD simulations for investigating conformational dynamics, nucleation, target-drug interactions, and other relevant molecular phenomena^39–51^.

### Dimensionality reduction (LDA) significantly improves neighborhood similarity

As discussed in the first subsection, neighborhood similarity evaluated using Euclidean distance can be incorrect and may lead to poor explanations. Here, we perform experiments to demonstrate the advantages of LDA-based similarity measure. Figure 4h shows that the LDA projection successfully generated two clusters of data points belonging to the in-explanation (predicted class of the instance requiring explanation) and not in-explanation classes (all other classes except predicted class) respectively. These well-separated clusters help in computing meaningful and improved distance measure *d*. In Fig. 4i, j, we illustrate the robustness of an LDA implementation against noisy and correlated features and compare results with Euclidean similarity implementation. We generate pure white noise by drawing samples from a normal distribution ${{{\mathcal{N}}}}(0,1)$ and generate correlated data by taking ${a}_{i}{x}_{i}+b{{{\mathcal{N}}}}(0,1)$ (e.g., *a*_*i*_ = 1.0, *b* = 0.2), where *x*_*i*_ are standardized features from the actual data. As shown in Fig. 4i, j, we construct synthetic neighborhoods by combining actual data from the four dihedral angles and adding one pure noise, four pure noise, and four correlated features, respectively. Since the synthetic features do not contain any information, their addition should not change similarity. Thus, we can compare the robustness of a measure by computing the average change in similarity per datapoint squared, which we call similarity error, *ΔΠ* ∈ [0, 1], as shown in Equation (9).

9 $$\Delta \Pi=\frac{1}{N}\mathop{\sum }_{i=1}^{N}{\left({\Pi }_{i}^{o}-{\Pi }_{i}^{s}\right)}^{2}$$

Here, the superscripts *o* and *s* represent similarities corresponding to the original and synthetic data points, respectively. We can see that LDA-based similarity performs significantly better in 100 independent trials compared to Euclidean similarity. On the other hand, the addition of one pure noise introduces a significant similarity error for the Euclidean measure. Thus we conclude that adopting LDA over Euclidean similarity measure produced a significantly improved explanation.

### Application to image classification: vision transformers (ViTs)

Transformers are a type of machine learning model characterized by the presence of self-attention layers and are commonly used in natural language processing (NLP) tasks^59^. The more recently proposed Vision transformers (ViTs)^37^ aim to directly apply the transformer architecture to image data, eliminating the need for convolutional layers, and have become a popular choice in computer vision. Per construction, ViTs are black-box models, and because of their practical usage, it is desirable to employ an explanation scheme to validate their predictions before deploying them.

ViTs operate by segmenting input images into smaller patches, treating each patch as a token similar to words in NLP. These patches are then embedded (patch-embeddings) and passed to the transformer layers conducting self-attention and feedforward operations. Such a design allows ViTs to capture long-range spatial dependencies within images and learn meaningful representations. Interestingly, ViTs are known to perform poorly with limited training data, but with sufficiently large datasets, ViTs have been shown to outperform convolutional layer-based models. Thus a typical ViT implementation includes two stages: first a large dataset is used to learn meaningful representation and pre-train a transferable model, followed by fine-tuning for specific tasks.

**Fig. 5:**
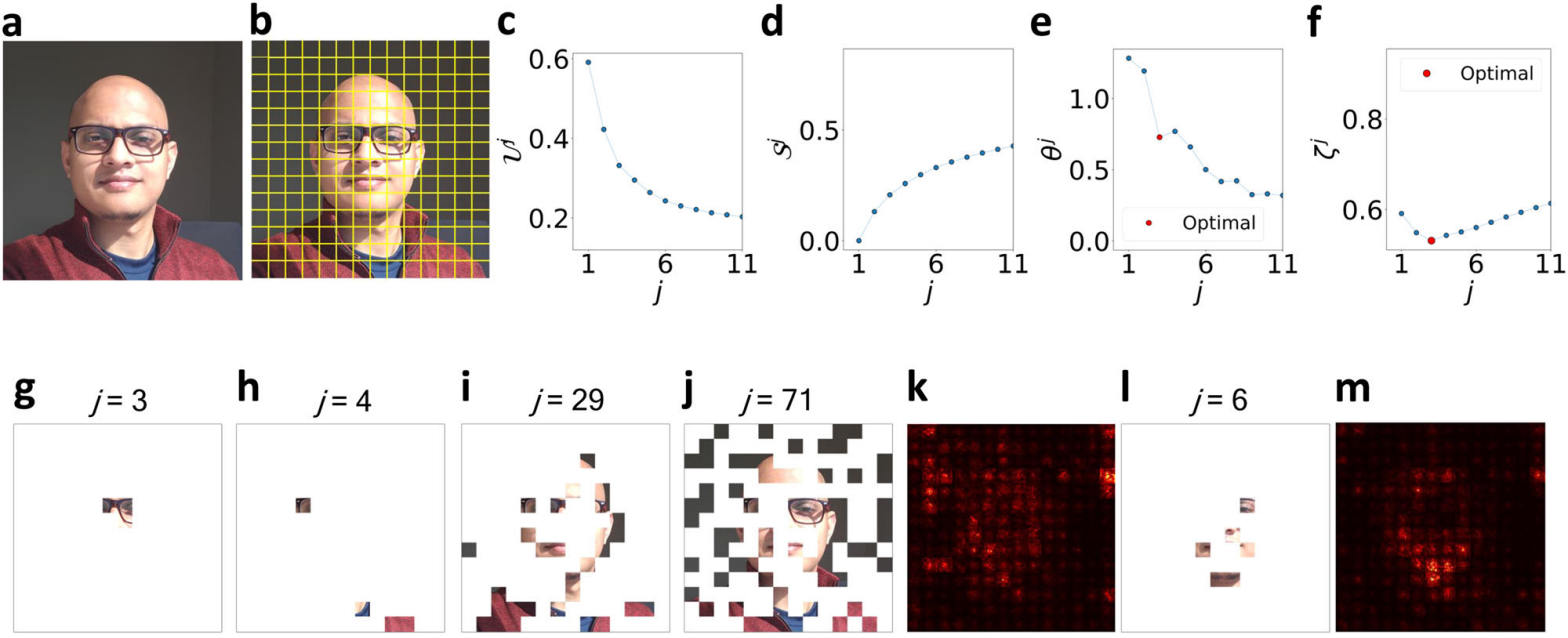
Using TERP to explain and check the reliability of a ViT trained on CelebA dataset. **a** ViT predicts the presence of 'Eyeglasses' in this image with a probability of 0.998. **b** Superpixel definitions for the test image following the 16 × 16 pixel definition of ViT patches. TERP results showcasing **c**${{{{\mathcal{U}}}}}^{\, j}$, **d**${{{{\mathcal{S}}}}}^{\, j}$, **e***θ* ^*j*^, and **f***ζ* ^*j*^ as functions of *j*, **g** corresponding TERP explanation. We can see the maximal drop in *θ* ^*j*^ happens when going from *j* = 2 to *j* = 3. By defining the optimal temperature ${\theta }^{o}=\frac{{\theta }^{\, \, j=2}+{\theta }^{\, \, j=3}}{2}$ as discussed in the “Results” section, a minimum in *ζ* ^*j*^ is observed at *j* = 3. Panels **h**–**j** show sanity checks^63^, i.e., the result of an AI explanation scheme should be sensitive under model parameter randomization (**h**), (**i**) and data randomization (**j**). **k** Saliency map results as baseline explanation for ‘Eyeglasses’ prediction. Red color highlights pixels with high absolute values of the class probability gradient across RGB channels. The high gradient at pixels not relevant to ‘Eyeglasses’ shows the limitation of the saliency map explanation. **l** TERP, and **m** saliency map explanations for the class ‘Male’. ${{{{\mathcal{U}}}}}^{\, j}$, ${{{{\mathcal{S}}}}}^{\, j}$, *ζ* ^*j*^, and *θ* ^*j*^ as functions of *j* for (**l**, **m**) are provided in the SI.

In this work, we employ a ViT pre-trained on the ImageNet-21k dataset from the authors^37,60,61^ and then fine-tune the model for predicting human facial attributes by training on the publicly available Large-scale CelebFaces Attributes (CelebA)^62^ dataset. CelebA is a large collection of 202,599 human facial images and each image is labeled with 40 different attributes (e.g., ‘Smiling’, ‘Eyeglasses’, ‘Male’, etc.). During training, input images are converted into 16 × 16 pixel patches resulting in a total of 196 patches for each CelebA image (224 × 224 pixel) depicted in Fig. 5b. Other details of the architecture and training procedure are provided in “Methods.”

To explain the ViT prediction ‘Eyeglasses’ (prediction probability of 0.998) for the image shown in Fig. 5a using TERP, we first construct human-understandable representative features by dividing the image into 196 superpixels (collection of pixels) corresponding to the 196 ViT patches as shown in Fig. 5b. Afterward, a neighborhood of perturbed images was generated by averaging the RGB color of randomly chosen superpixels following the neighborhood generation scheme outlined in “Methods.” Figure 5c–f shows ${{{{\mathcal{U}}}}}^{\, j}$, ${{{{\mathcal{S}}}}}^{\, j}$, *θ* ^*j*^, and *ζ* ^*j*^ as functions of *j* after implementing the TERP protocol (Fig. 3). Thus, TERP explanation enables us to conclude that the ViT prediction of ‘Eyeglasses’ was made for the correct reasons. The optimal TERP explanation shown in Fig. 5g appears at *j* = 3, due to the maximal decrease in *θ* ^*j*^ as *j* is increased from 2 to 3. Using Equations (7) and (8), *ζ* ^*j*^ is calculated, and a minimum occurs at *j* = 3.

### Data and model parameter randomization experiments show TERP explanations are sensitive

To establish that TERP indeed takes both the input data and the black-box model into account when generating explanations, we subject our protocol to the sanity tests developed by Adebayo et al.^63^. We achieve this by taking the fine-tuned ViT model and randomizing the model parameters in a top-to-bottom cascading fashion following their work and obtaining corrupted models. Specifically, we randomize all parameters of ViT blocks 11 − 9 and blocks 11 − 3, respectively, to obtain two corrupt models. TERP explanations for ‘Eyeglasses’ for these two models are shown in Fig. 5h–i. Plots showing ${{{{\mathcal{U}}}}}^{\, j}$, ${{{{\mathcal{S}}}}}^{\, j}$, *ζ* ^*j*^, and *θ* ^*j*^ as functions of *j* for these models are provided in the SI (Supplementary Fig. S2). Here, the idea is that, due to randomization, the explanation will not match the ground truth. However, a good AI explanation scheme should be sensitive to this randomization test and produce different explanations from the fully trained model. Similarly, we implemented the data randomization test (Fig. 5j) proposed in the same work, where the labels of the training data are randomized prior to training, and a new ViT is obtained (training details provided in the SI) using the corrupted data. Again, the results of an AI explanation method should be sensitive to this randomization. From the corresponding TERP explanations shown in Fig. 5h–j, we conclude TERP passes both randomization tests.

### Baseline benchmark against saliency map shows TERP explanations are reliable

To understand the validity, robustness, and human interpretability of the explanations, we benchmarked TERP against saliency map, LIME, and SHAP, respectively. In this section, we first show that TERP explanations are significantly better, and reasonable compared to a baseline method, i.e., a simple gradient-based saliency map (additional details in “Methods”) for ‘Eyeglasses’ prediction using the previously trained ViT. Comparison with more advanced methods (LIME, and SHAP) to demonstrate how our work contributes to the existing field is discussed in the next subsection.

From Fig. 5k, we see the limitations of the saliency explanation, e.g., a lot of pixels irrelevant to ‘Eyeglasses’ are detected to have high absolute values of the probability gradient across the RGB channels. This is not surprising since saliency maps are known to detect color changes, object edges, and other high-level features instead of learning a relationship between model inputs and class prediction^63^. We also generated TERP and saliency map explanations for the label ‘Male’ as shown in Fig. 5l, m (further details in the SI). Again, the saliency map explanation includes pixels that should be irrelevant for this predicted class. Contrarily, TERP explanations involve pixels that should be relevant to the respective classes demonstrating the validity of the results.

### Comparison with advanced methods demonstrates TERP explanations are unique

In this subsection, we compare TERP with state-of-the-art methods for generating unique and highly human-interpretable explanations. To ensure a fair comparison, we focus on other widely used model-agnostic, post-hoc explanation schemes (LIME^19^, and SHAP^20^) that work only on the input and output layers of a black-box model.

LIME generates local, linear approximation (*f*) to black-box predictions (*g*) by minimizing: $\xi (\,{\mbox{x}})={\mbox{argmin}}\,f{{{\mathcal{L}}}}(g,f,{\pi }_{{{{\rm{x}}}}})+\Omega (f)$, where ${{{\mathcal{L}}}}$ is a fidelity function (typically root-mean-squared error), *π*_x_ is neighborhood similarity, and *Ω* is the complexity measure of the surrogate linear model. In practice, LIME is implemented by first performing weighted linear regression and then either (1) selecting the top *j* features with extreme coefficients, or (2) by directly implementing Lasso regression with *L*1 regularization^64^ for constructing sparse models, where the degree of sparsity can be tuned by a hyperparameter *α*. Both *j* and *α* typically depend on the instance under investigation and will need to be set to a reasonable value by the user. Thus, an accurate human interpretability-based mechanism for generating unique explanations is missing in LIME, and when analyzing a large number of black-box predictions, significant testing/human intervention becomes necessary.

While both TERP and LIME use similar fidelity functions, the main difference is that TERP does not use model complexity or simplicity as a proxy for human interpretability. As discussed in the “Introduction”, such metrics can be misleading, and TERP directly computes the degree of human interpretability by introducing the concept of interpretation entropy. Afterward, a unique explanation is generated by identifying the set of features causing the highest decrease in unfaithfulness per unit increase in entropy.

**Fig. 6:**
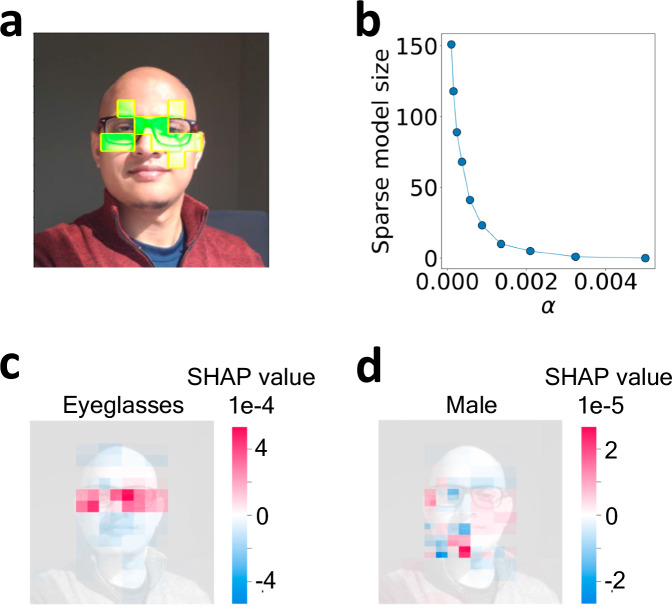
Black-box explanations for state-of-the-art approaches. **a** LIME explanation for ‘Eyeglasses’ with top *j* = 10 features, **b** Sparse model size vs. hyperparameter *α* that regulates the strength of *L*1 regularization. SHAP values for **c** ‘Eyeglasses’, **d**, and ‘Male’ prediction respectively. Consistency of these results with explanations shown in Fig. 5 validates TERP.

We applied LIME to explain the ViT prediction for ‘Eyeglasses’, and in Fig. 6a, the top 10 features contributing to the prediction are shown. We also implemented the second approach in LIME, i.e., Lasso regression for sparse models for 10 different values of *α*. As *α* is increased, the number of selected features in the explanation decreases, as shown in Fig. 6b. While the relevant superpixels identified by LIME are reasonable and overlap with the superpixels identified by TERP (Fig. 5g), LIME involves hyperparameter selection/human intervention which can be unfeasible for high-throughput experiments, e.g., when analyzing MD data.

After LIME, we implemented another widely used state-of-the-art method, SHAP, for explaining ‘Eyeglasses’, and ‘Male’ predictions as shown in Fig. 6c, d. A feature associated with an extreme SHAP value indicates a high contribution to black-box prediction. Specifically, the SHAP value associated with a feature *j* can be obtained using: ${\phi }_{j}={\sum }_{S}\frac{| S| !(N-| S| -1)!}{N!}[v(S\cup \{\, \, j\})-v(S)]$. Here, the prefactor represents the weight of the marginal contribution (enclosed in []) of feature *j* to *S* where *S*, ∣*S*∣, and *N* represent a specific set of features (coalition), number of features in that specific coalition, and total number of features, respectively. The marginal contribution is evaluated by subtracting the effects of the feature *j* in predictions when *j* is present and absent in the coalitions respectively. After obtaining SHAP values for all the features, a sparse explanation is typically obtained by taking the top *j* (*j* is user-defined) features with the most extreme SHAP values. Thus, similar to LIME, SHAP explanations are also not unique. By comparing SHAP results with TERP (Fig. 5g, l), we again see that the relevant features overlap, which validates TERP explanation.

In this section, we compared TERP with two widely used state-of-the-art model-agnostic, post-hoc approaches and demonstrated the validity of TERP explanations. Furthermore, by employing the theory developed in this work, TERP successfully generated highly human-interpretable, unique explanations, unlike the established methods. Implementation details of LIME and SHAP are provided in “Methods.”

### Application to text classification: attention-based bidirectional long short-term memory (Att-BLSTM)

Classification tasks in natural language processing (NLP) involve identifying semantic relations between units appearing at distant locations in a block of text. This challenging problem is known as relation classification, and models based on long short-term memory (LSTM)^65^, gated recurrent unit (GRU)^66^, and transformers^59^ have been very successful in addressing such problems. In this work, we look at the widely used attention-based bidirectional long short-term memory^38^ (Att-BLSTM) classifier and apply TERP to explain its predictions.

First, we trained an Att-BLSTM model on Antonio Gulli’s (AG’s) news corpus^67^, which is a large collection of more than 1 million news articles curated from more than 2000 news sources. The labels associated with each news article in the dataset indicate the section of the news source (e.g., World, Sports, Business, or Science and technology) that the news was published in. Afterward, we employed the trained model and obtained prediction for a story titled “AI predicts protein structures,” published in ‘Nature’s biggest news stories of 2022’^68^.

To implement TERP for probing a black-box prediction involving text input (sequence of sentences), first, the text is passed through a tokenizer (nltk^69^) which generates a dictionary of words/phrases contained in that text. These words are the representative features to be used in TERP. Afterward, a neighborhood of the perturbed text is generated by randomly choosing and removing all instances of different words from the text. TERP processes the neighborhood as numerical values for linear model construction by creating a one-hot-encoded matrix where the columns represent the presence or absence of the different words in the perturbed text.

**Fig. 7:**
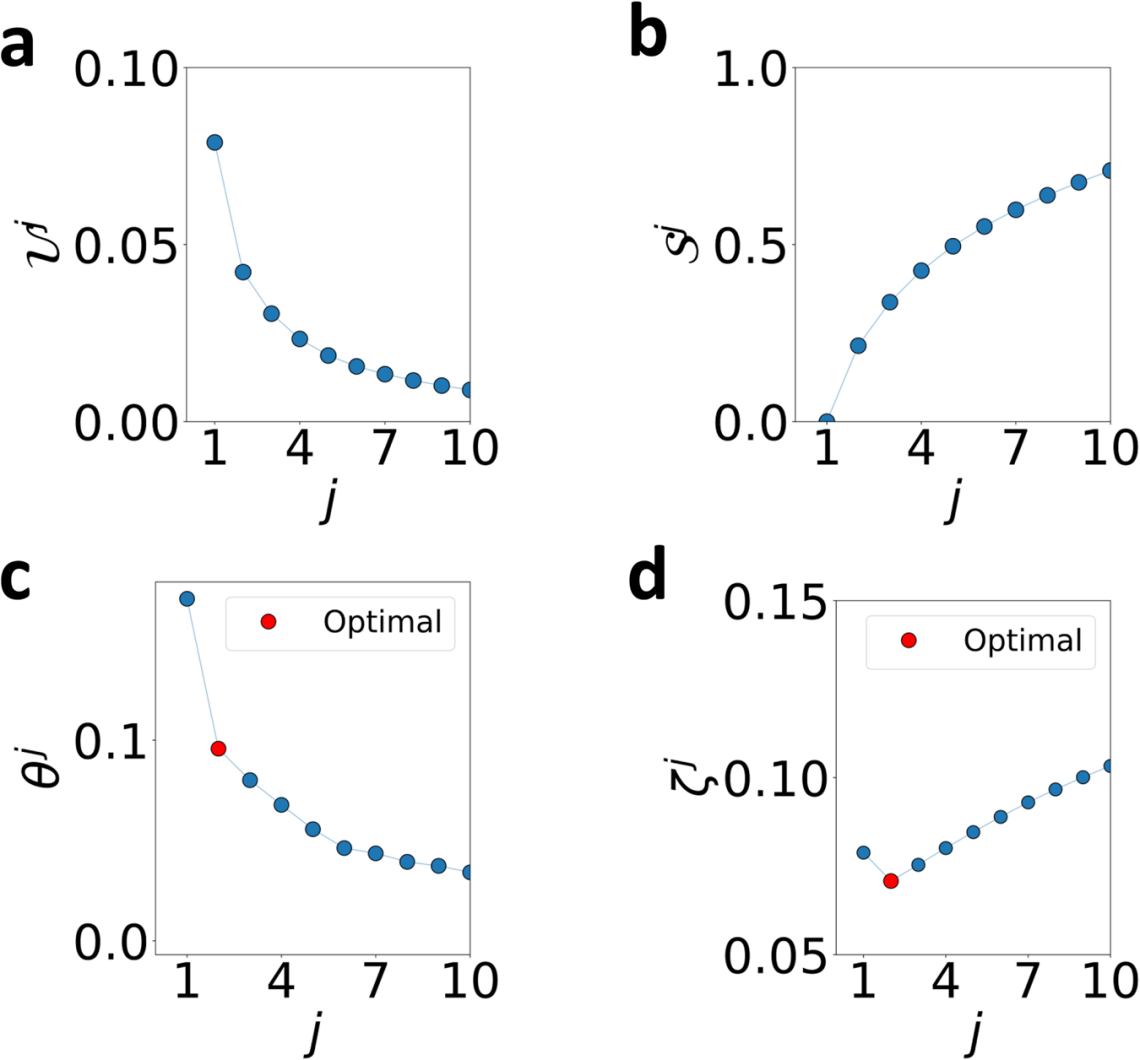
Using TERP to explain and check the reliability of Att-BLSTM model trained on AG’s news corpus to predict the news story titled “AI predicts protein structures”. **a**${{{{\mathcal{U}}}}}^{\, j}$ vs. *j*, **b**${{{{\mathcal{S}}}}}^{\, j}$ vs. *j*, **c***θ* ^*j*^ vs. *j*, **d** *ζ* ^*j*^ vs. *j* plots showing the optimal explanation appears at *j* = 2, due to the maximum drop in *θ* ^*j*^ as *j* is increased from 1 to 2.

As a specific instance, the Att-BLSTM classifier predicted that the story titled “AI predicts protein structures” is about Science and Technology, and we implemented TERP to generate the optimal explanation behind this prediction as shown in Fig. 7. Here, the maximum decrease in *θ* ^*j*^ occurs when going from *j* = 1 to *j* = 2 and thus, *ζ* ^*j*^ has a minimum at *j* = 2. The most influential keywords were identified to be ‘species’, and ‘science’ with *p*_*k*_ = 0.47, and 0.53 respectively. This gives confidence that the Att-BLSTM model was able to classify the news story for the correct reasons.

## Discussion

The widespread adoption of AI-based black-box models has become a standard practice across various fields due to their ability to be deployed without requiring an in-depth understanding of the underlying processes. However, this advantage also poses challenges regarding trustworthiness and the explanation of AI models. In this study, we introduce a thermodynamics-inspired framework to create interpretable representations of complex black-box models. Our objective was to find representations that minimize discrepancies from the true model while remaining highly interpretable to humans using a concept similar to the energy-entropy trade-off. Furthermore, the concept of interpretation entropy introduced in this work has the potential to be useful in general human interpretability-based model selection beyond ML. In future work, efficient optimization algorithms can be developed for general-purpose linear regression that uses Equation (4) as a regularization to directly construct human-interpretable models.

We showcased the effectiveness of this approach in various AI applications, including image classification, text analysis, and molecular simulations. While several methods^17,20,70,71^ have been proposed to address AI interpretability in the past, only a handful, such as refs. ^72–77^, have been utilized to elucidate molecular simulations. Importantly, our work marks one of the pioneering applications of interpretability techniques in the rapidly evolving field of AI-enhanced molecular dynamics.

Recent applications of our framework (TERP), have been instrumental in uncovering key mechanisms behind crystal nucleation^78^ and hydrophobic ligand dissociation^79^. Given the critical role of molecular sciences in uncovering chemical reaction pathways^80^, understanding disease mechanisms^81^, designing effective drugs^82^, and numerous other vital areas, it is crucial to ensure accurate analysis, as errors in black-box models can have significant financial and public health implications. TERP should provide practitioners of molecular sciences a way to explain these black-box models on a footing made rigorous through simple yet powerful parallels with the field of thermodynamics.

## Methods

### Neighborhood generation

We take inspiration from the work of Ribeiro et al.^19^ and generate a single instance of the perturbed sample around the neighborhood of an instance **x** with *n* features by first drawing *n* numbers from a uniform distribution, {*t*_1_, *t*_2_, …, *t*_*n*_} ∈ [0, 1]. The *i*th feature is perturbed if *t*_*i*_ ≥ 0.5; otherwise, the feature is kept unchanged. Once a feature is chosen for perturbation, the specific scheme for obtaining perturbed values depends on the corresponding data type.

For tabular data, if a feature *x*_*i*_ is continuous, it is updated by *x*_*i*_ = *x*_*i*_ + *ϵσ*_*i*_ where *σ*_*i*_ is the standard deviation of the feature in the training data and *ϵ* is a small noise drawn from a Gaussian distribution. For categorical data, feature value *x*_*i*_ is updated by ${x}_{i}=x{\prime}$, where $x{\prime}$ is sampled from the training data. For text, an instance is first converted into tokens^83^, which are considered as features. If a token is chosen for perturbation by following the scheme described above, it is replaced by a new token sampled from training data. For images, superpixels are defined and, if chosen for perturbation, are updated by averaging the colors of all the pixels within that particular superpixel. If the input data contains a high number of features, a strategy discussed in Supplementary Note 4 can be adopted for an efficient implementation of TERP.

### AI-augmented MD method: VAMPnets

The molecular system for alanine dipeptide in vacuum was parametrized using the forcefield CHARMM36m^84^ and prepared using CHARMM-GUI^85^. A 100 ns MD simulation of alanine dipeptide in vacuum at 450 K temperature and 1 atm pressure was performed using Nose-Hoover thermostat and Parrinello-Rahman barostat^86,87^ in GROMACS^88^.

A VAMPnets^36^ deep neural network was constructed from two identical artificial neural network lobes, that take trajectory order parameters (OPs) at time steps *t* and *t* + *τ*, respectively, as inputs. The input data was passed through several layers of neurons, and finally, a VAMP-2 score was calculated by merging results from the outputs of both lobes. The neural network model parameters were tuned in successive iterations that maximize the VAMP-2 score (Supplementary Fig. S3). In this way, a Markov state model at a specific lagtime *τ* can be learned that describes the slow processes of interest.

In this work, the VAMPnets implementation was performed using the PyEMMA^89^ 2.5 and Deeptime^90^ 0.4.2 Python libraries by constructing the neural network architecture depicted in Supplementary Fig. S4. Other training hyperparameters are: *τ* = 0.05ps, learning rate = 0.0005, epochs = 50.

### Image classification: vision transformers (ViTs)

Large-scale CelebFaces Attributes (CelebA) Dataset^62^ contains 202,599 celebrity images, each annotated with 40 binary attributes. CelebA offers the dataset in two different formats: (1) actual raw images and (2) processed data with aligned facial images. In this work, we employed the latter and divided the dataset into training, validation sets with a ratio of 50: 50. The training data was then used to train a ViT model.

The model was trained until validation metrics (f1 score) did not improve for 5 consecutive epochs using a learning rate of 0.00001. The model with the highest validation metric was saved as the trained model (Supplementary Fig. S5).

Training and inference using ViT was implemented using PyTorch-lightning 1.5 and Python 3.9. The pre-trained ViT model was pulled from the timm Python library. For saliency analysis, the absolute values of the gradients of prediction probabilities with respect to input pixels were calculated using the *backward()* method of PyTorch during a backward pass.

The authors affirm that human research participants provided informed consent for publication of the images in Figs. 5 and 6.

### Implementation details for LIME, and SHAP

Both LIME (0.2.0.1) and SHAP (0.46.0) were implemented in Python. The chosen hyperparameters for LIME: number of samples = 5000, LASSO(maximum iterations) = 1000, and SHAP number of evaluations = 1000. LIME was implemented by using the same superpixel definitions that were used for TERP explanation to ensure a fair comparison. To generate a perturbed image in SHAP, patches of 14 × 14 pixels were systematically blurred for various coalitions.

### Text classification: attention-based bidirectional long short-term memory (Att-BLSTM)

In this work, we employed Python implementation of Att-BLSTM^38^ obtained from github.com/Renovamen/Text-Classification with pre-trained GloVe word embedding. Att-BLSTM model was trained on Antonio Gulli’s (AG’s) news corpus^67^ for 10 epochs, finally reaching a validation accuracy of 92.0%.

## Supplementary information

Supplementary Information

Peer Review File

## Source data

Source data

## Data Availability

The data that support the findings of this study are openly available. The AG’s news corpus dataset was obtained from ref. ^67^, and CelebA dataset from ref. ^62^ in accordance with the Terms of Service of the respective web resources. The molecular dynamics trajectory of alanine dipeptide and the trained black-box models used in this study have been deposited in the Figshare database under accession code https://figshare.com/articles/dataset/Black-box_models_for_TERP_interpretation/24475003^91^. Underlying data for all the plots/graphs are provided in a Source Data file. Source data are provided with this paper.

## Code availability

Python implementation of TERP for explaining black-box predictions is available at github.com/tiwarylab/TERP^92^.
