# Supplementary material for: Thermodynamics-inspired explanations of artificial intelligence
Source: Nat Commun. 2024 Sep 9;15:7859. doi: 10.1038/s41467-024-51970-x (PMC11385982; doi:10.1038/s41467-024-51970-x)
Supplement: Supplementary file 1 — Supplementary Information [file 41467_2024_51970_MOESM1_ESM.pdf]

Supplementary Information: Thermodynamics-inspired Explanations of Artificial Intelligence

Shams Mehdi[1] **and Pratyush Tiwary**[2,3,*]

[1]Biophysics Program and Institute for Physical Science and Technology, University of Maryland, College Park 20742, USA

[2]Department of Chemistry and Biochemistry and Institute for Physical Science and Technology, University of Maryland, College Park 20742, USA

[3]University of Maryland Institute for Health Computing, Bethesda, Maryland 20852, USA

[*] Electronic mail: ptiwary@umd.edu

SUPPLEMENTARY NOTES

Supplementary Note 1: $\mathcal{S}^j$ is a monotonically increasing function of the number of features with non-zero coefficients (j)

Let linear regression with j features yields j non-zero coefficients ($\{f_1, f_2, ..., f_j\}|1 < j \leq n$), where total number of features under consideration is n. As discussed in the main text, we can define a probability distribution $p_k := \frac{|f_k|}{\sum_{i=1}^{n}|f_i|}$ and interpretation entropy ($\mathcal{S}^j$),

$$\mathcal{S}^j(p) = \frac{-1}{\log\, n}\sum_{k=1}^{n} p_k \, \log\, p_k = \frac{-1}{\log\, n}\sum_{k=1}^{j} p_k \, \log\, p_k \tag{1}$$

Similarly, interpretation entropy for a $j+1$ coefficient model is given by,

$$\mathcal{S}^{j+1}(p') = \frac{-1}{\log\, n}\sum_{k=1}^{n} p'_k \, \log\, p'_k = \frac{-1}{\log\, n}\sum_{k=1}^{j+1} p'_k \, \log\, p'_k \tag{2}$$

$\mathcal{S}^j$ is a monotonically increasing function iff,

$$[\mathcal{S}^{j+1} - \mathcal{S}^j](\log\, n) \geq 0 \tag{3}$$

Now, $(\log\, n)\mathcal{S}^{j+1} = -\sum_{k=1}^{j} p'_k \, \log\, p'_k - p'_{j+1}\, \log\, p'_{j+1}$. If the features are independent, adding new features will not change the relative ratios of the existing features i.e., $\frac{p_k}{p'_k} = \alpha$. In other words, we need to rescale the existing distribution by a single positive constant α to preserve the relative ratios, since $\sum_{k=1}^{j} p_k = \sum_{k=1}^{j+1} p'_k = 1$. Now,

$$\begin{aligned}
\sum_{k=1}^{j+1} p'_k &= 1 \\
\sum_{k=1}^{j} p'_k + p'_{j+1} &= 1 \\
\sum_{k=1}^{j} \frac{p_k}{\alpha} + p'_{j+1} &= 1 \\
\frac{1}{\alpha} * 1 + p'_{j+1} &= 1 \\
p'_{j+1} &= 1 - \frac{1}{\alpha}
\end{aligned} \tag{4}$$

Now,

$$
\begin{aligned}
(\log\, n)\mathcal{S}^{j+1} &= -\sum_{k=1}^{j} \frac{p_k}{\alpha}\, \log \frac{p_k}{\alpha} - p'_{j+1}\, \log p'_{j+1} \\
= -\sum_{k=1}^{j} \frac{p_k}{\alpha}\, \log p_k + \sum_{k=1}^{j} \frac{p_k}{\alpha}\, \log \alpha &- (1-\frac{1}{\alpha})\, \log\,(1-\frac{1}{\alpha}) \\
&= \frac{\log\, n}{\alpha}\mathcal{S}^{j}(p) + \frac{1}{\alpha}\, \log \alpha - (1-\frac{1}{\alpha})\, \log\,(1-\frac{1}{\alpha})
\end{aligned} \tag{5}
$$

$$
\implies [\mathcal{S}^{j+1}(p') - \mathcal{S}^{j}(p)](\log\, n) = (\frac{1}{\alpha} - 1)(\log\, n)\mathcal{S}^{j}(p) + \frac{1}{\alpha}\, \log \alpha - (1-\frac{1}{\alpha})\, \log\,(1-\frac{1}{\alpha}) \tag{6}
$$

The strategy here is to rewrite the R.H.S of the above equation completely in terms of the parameter α. Now, let's check the lower limit of α. Since, the minimum of p'_{j+1} is zero,

$$
\begin{aligned}
p'_{j+1} &\geq 0 \\
\frac{\alpha - 1}{\alpha} &\geq 0 \\
\implies \alpha &\geq 1
\end{aligned} \tag{7}
$$

To check the upper limit of α we recognize that the probability (p'_{j+1}) of the newly added feature is less than or equal to the average of the existing probabilities.

$$
\begin{aligned}
p'_{j+1} &\leq \frac{\sum_k p_k}{j} \\
p'_{j+1} &\leq \frac{1}{j} \\
\frac{\alpha-1}{\alpha} &\leq \frac{1}{j} \\
\implies \alpha \leq \frac{j}{j-1} \implies j &\leq \frac{\alpha}{\alpha - 1}
\end{aligned} \tag{8}
$$

Now, the maximum value that the first term of R.H.S. of Supplementary Equation (6) can take is when the probability distribution (p) is uniform. Using Jensen's inequality [1], $\mathcal{S}^{j}(p) \leq \log j$. Thus,

$$
\implies [\mathcal{S}^{j+1}(p') - \mathcal{S}^{j}(p)](\log\, n) \geq (\frac{1}{\alpha} - 1)(\log\, n)(\log\, j) + \frac{1}{\alpha}\, \log \alpha - (1-\frac{1}{\alpha})\, \log\,(1-\frac{1}{\alpha}) \tag{9}
$$

Substituting the upper limit of j as found in Supplementary Equation (8) in the above expression we get,

$$\begin{aligned}[\mathcal{S}^{j+1}(p') - \mathcal{S}^{j}(p)](\log n) \geq (\frac{1}{\alpha} - 1)(\log n) \log \frac{\alpha}{\alpha - 1} + \frac{1}{\alpha} \log \alpha - (1 - \frac{1}{\alpha}) \log (1 - \frac{1}{\alpha}) \\ \implies [\mathcal{S}^{j+1}(p') - \mathcal{S}^{j}(p)](\log n) \geq \frac{1}{\alpha} \log \alpha\end{aligned} \tag{10}$$

Since α is a positive constant, this shows that $\mathcal{S}^j$ increases monotonically with j, if the features are independent. When the rescaling factor $\alpha = 1$ i.e., when no additional feature is added the R.H.S. of the above expression becomes zero.

Supplementary Note 2: $\mathcal{S}$ monotonically increases as $\mathcal{U}$ decreases

Since, the linear regressions are performed with standardized data, if the probability (p_k) of a feature is relatively high, the feature is understood to have a high contribution towards predictions. However, a high probability value makes the distribution less sharply peaked i.e., high $\mathcal{S}$ compared to a low probability value. Thus, in practice $\mathcal{S}$ monotonically increases as $\mathcal{U}$ decreases.

An illustrative $\mathcal{U}$ vs. $\mathcal{S}$ profile is shown in Supplementary Fig. S1.

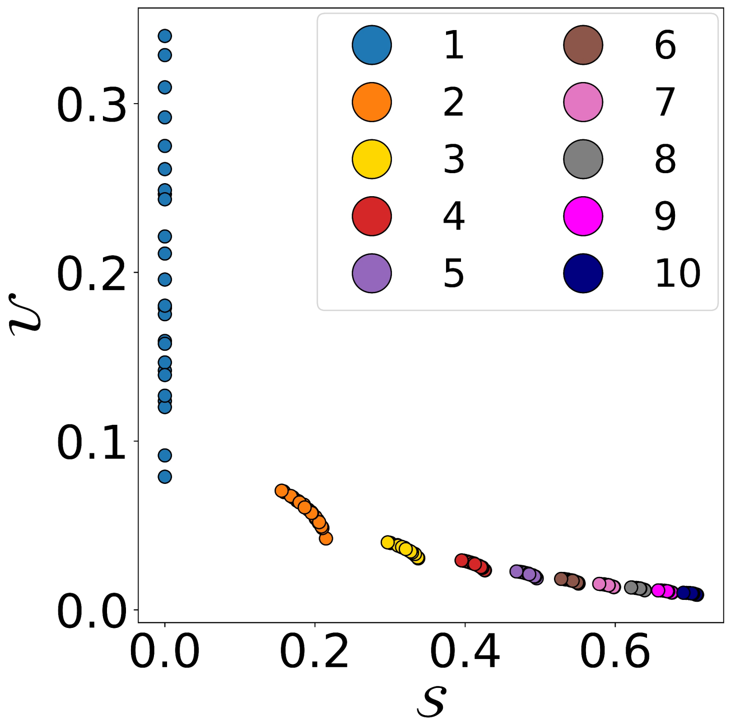

Supplementary Fig. S1: Illustrative $\mathcal{U}$ vs. $\mathcal{S}$ plot. The different colors correspond to the linear regression of $j = 1, 2, 3, 4, 5, 6, 7, 8, 9, 10$ coefficient models respectively.

Supplementary Note 3: Existence of a unique solution

The exact values of unfaithfulness $\mathcal{U}$, and interpretation entropy $\mathcal{S}$ functions depend on the nature of the problem/data. For practical problems, $\mathcal{U}$ plateaus with high j i.e., $\frac{\Delta\mathcal{U}}{\Delta j} \approx 0$, while $\mathcal{S}$ has a lower limit of $\frac{1}{\alpha}$ log α as shown in Supplementary Note 1. As a result, when θ is increased slowly from zero, $\frac{\Delta\mathcal{S}}{\Delta j}$ dominates $\frac{\Delta\mathcal{U}}{\Delta j}$ at high j, while at low j, $\frac{\Delta\mathcal{U}}{\Delta j}$ dominates $\frac{\Delta\mathcal{S}}{\Delta j}$. This phenomenon produces the desired global minima at the optimal j coefficient model at a specific temperature θ.

Supplementary Note 4: Strategy for analyzing high number of features

TERP implements a forward feature selection[2] through linear regression, and the number of analyzed models can increase greatly with the number of features. A simple strategy to address this is the implementation of a pre-processing round of linear regression to identify and discard near-zero coefficient features prior to implementing TERP.

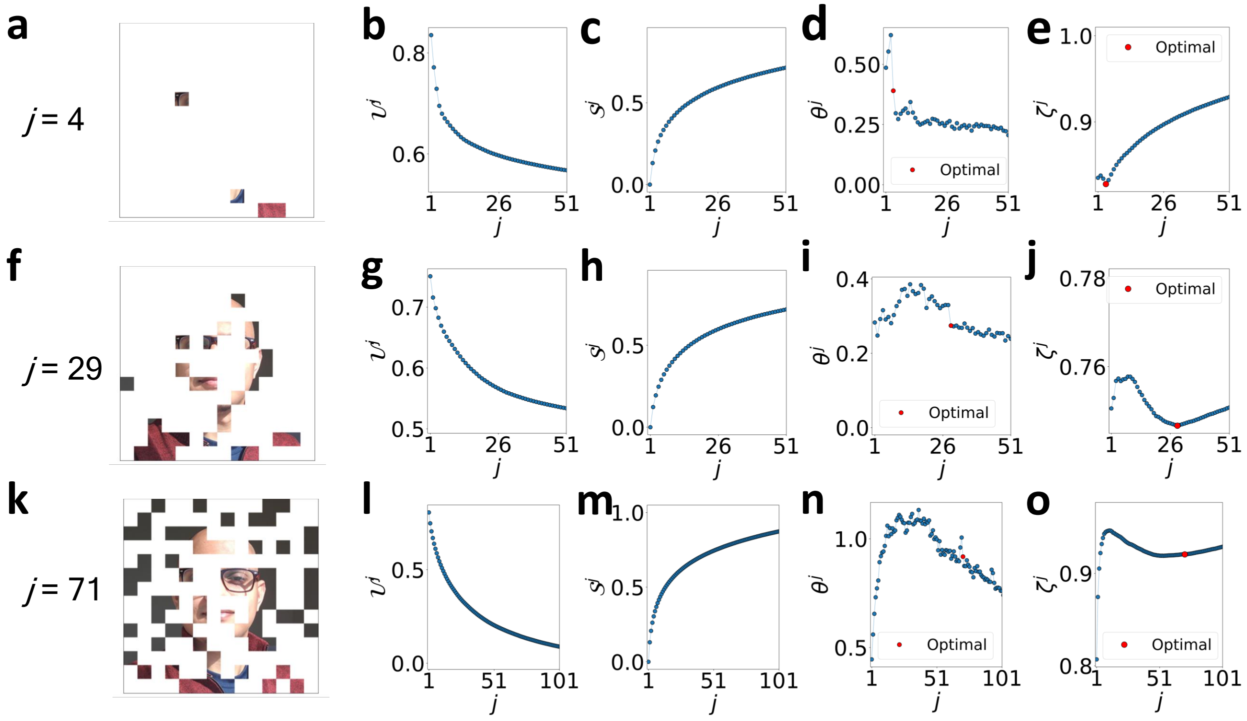

Supplementary Fig. S2: TERP results for parameter and data randomization sanity checks. For ViT[3] prediction 'Eyeglasses', panels **a-e** show TERP optimal explanation, $\mathcal{U}^j$, $\mathcal{S}^j$, $\theta^|$, and ζ^j when all the parameters in ViT architecture blocks $11 - 6$ were randomized i.e., drawn from a normal distribution. Panels **f-j** show results when all the parameters in ViT architecture blocks $11 - 3$ were randomized. Panels **k-o** show results for the data randomization test i.e., when a new ViT model is trained by randomizing all the labels associated with each sample in the training set during training.

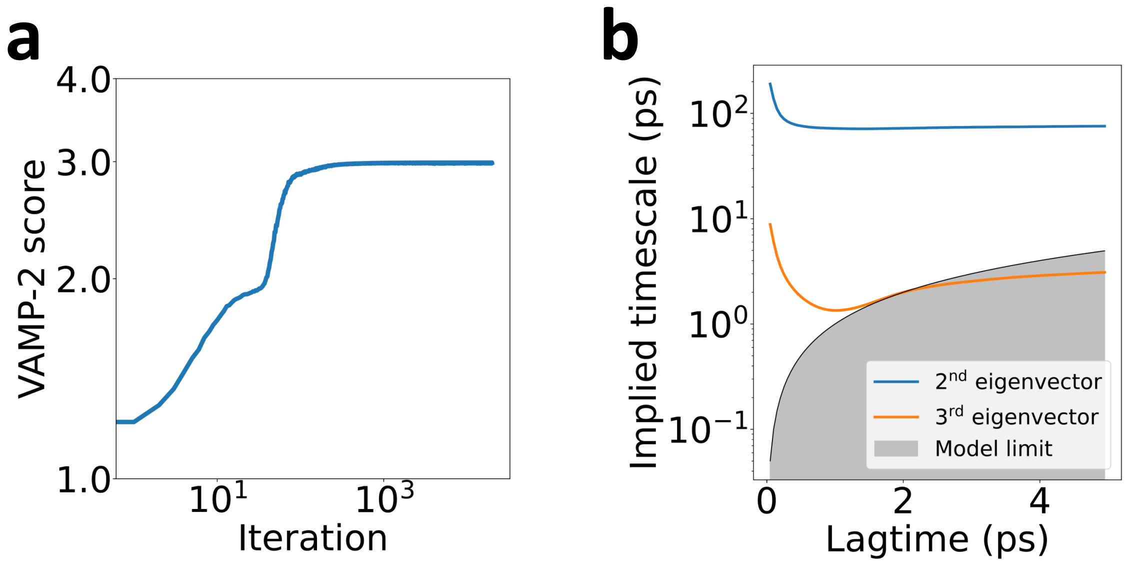

Supplementary Fig. S3: VAMPnets training summary. a Maximized VAMP-2 score for 50 training epochs. **b** Implied timescale vs. lagtime τ for second and third eigenvectors represented by blue and orange colors corresponding to transitions between different metastable states. Timescales within the grey region are faster than τ and cannot be resolved by the VAMPnets model.[4]

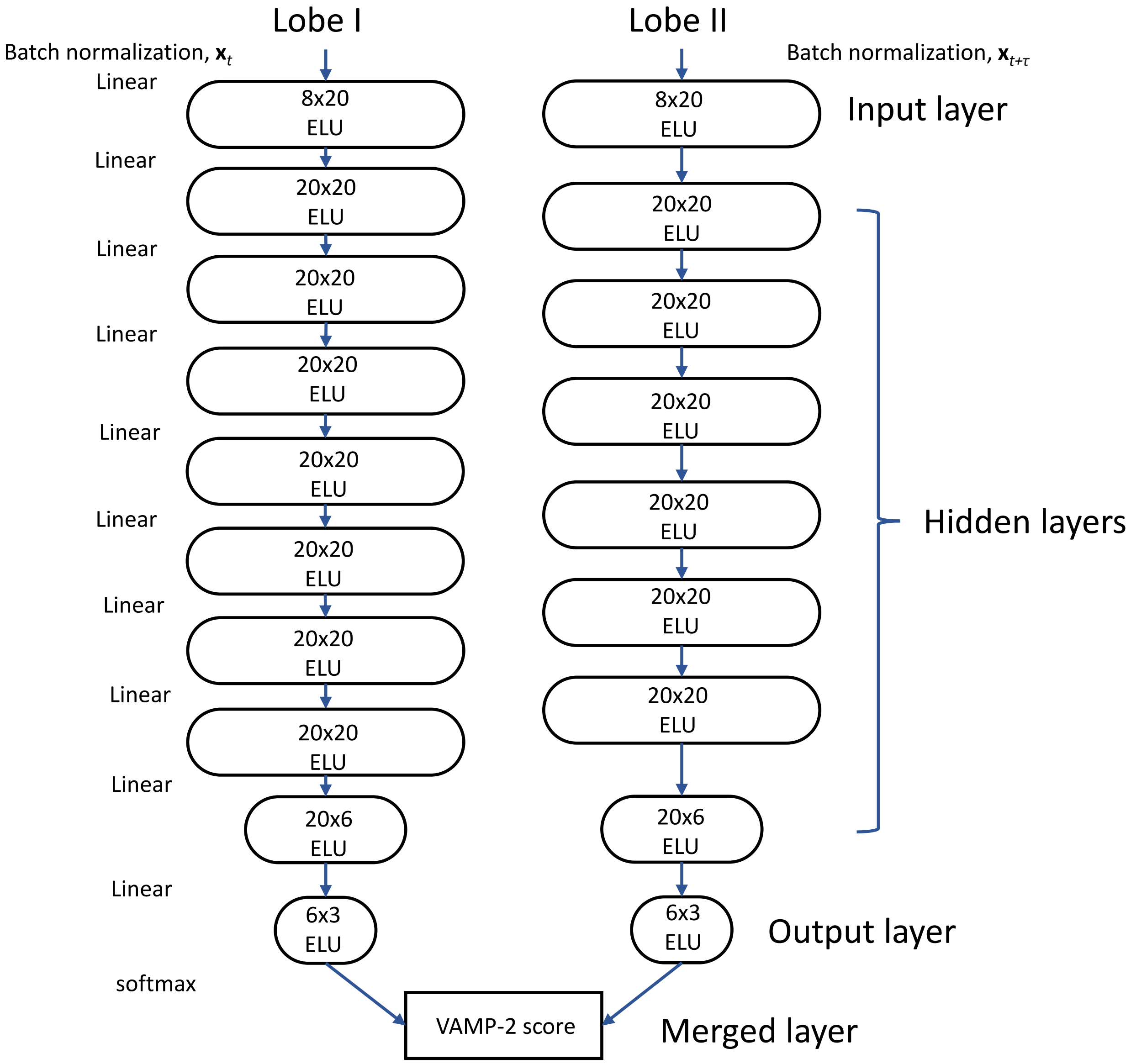

Supplementary Fig. S4: VAMPnets architecture.

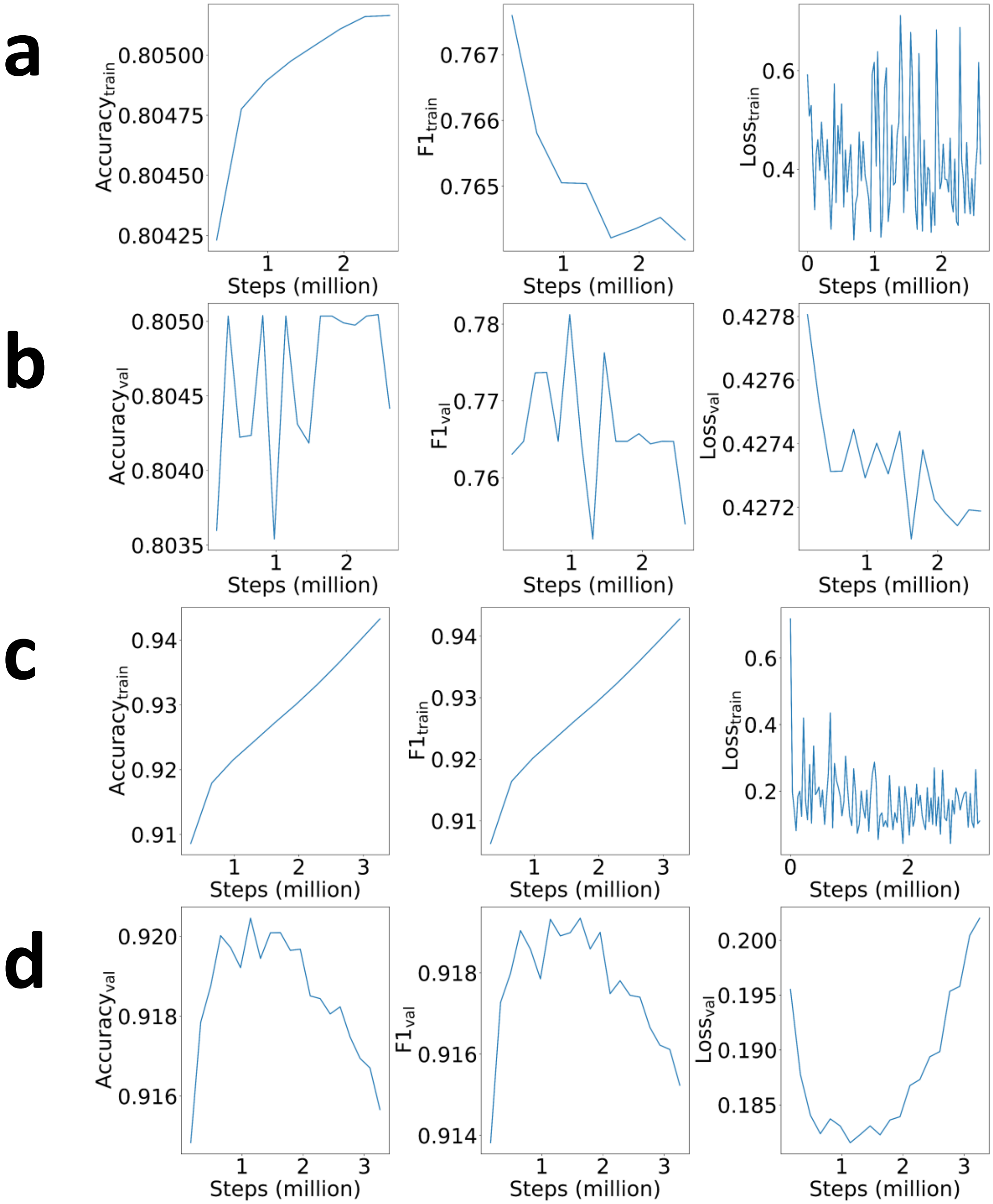

Supplementary Fig. S5: ViT model performance metrics (accuracy, f1 score, and binary cross entropy loss) as a function of model parameter updates. a Training, **b** validation of the data randomized model. **c** Training, **d** validation of the fine-tuned model. All the models were trained using a batch size of 1. Thus, during training, parameters were updated each time an image was passed to the model.

Supplementary References

[1] P. Bromiley, N. Thacker, and E. Bouhova-Thacker, Statistics and Inf. Series (2004-004) **9**, 2 (2004).

[2] A. Jović, K. Brkić, and N. Bogunović, in *2015 38th international convention on information and communication technology, electronics and microelectronics (MIPRO)* (Ieee, 2015) pp. 1200–1205.

[3] A. Dosovitskiy, L. Beyer, A. Kolesnikov, D. Weissenborn, X. Zhai, T. Unterthiner, M. Dehghani, M. Minderer, G. Heigold, S. Gelly, *et al.*, arXiv preprint arXiv:2010.11929 (2020).

[4] A. Mardt, L. Pasquali, H. Wu, and F. Noé, Nature communications **9**, 1 (2018).
