# Supplementary material for: Thermodynamics-inspired explanations of artificial intelligence
Source: Nat Commun. 2024 Sep 9;15:7859. doi: 10.1038/s41467-024-51970-x (PMC11385982; doi:10.1038/s41467-024-51970-x)
Supplement: Supplementary file 2 — Peer Review File [file 41467_2024_51970_MOESM2_ESM.pdf]

nature portfolio

Peer Review File

Thermodynamics-inspired Explanations of Artificial Intelligence

Open Access This file is licensed under a Creative Commons Attribution 4.0 International License, which permits use, sharing, adaptation, distribution and reproduction in any medium or format, as long as you give appropriate credit to the original author(s) and the source, provide a link to the Creative Commons license, and indicate if changes were made. In the cases where the authors are anonymous, such as is the case for the reports of anonymous peer reviewers, author attribution should be to 'Anonymous Referee' followed by a clear attribution to the source work. The images or other third party material in this file are included in the article's Creative Commons license, unless indicated otherwise in a credit line to the material. If material is not included in the article's Creative Commons license and your intended use is not permitted by statutory regulation or exceeds the permitted use, you will need to obtain permission directly from the copyright holder. To view a copy of this license, visit http://creativecommons.org/licenses/by/4.0/.

REVIEWER COMMENTS

Reviewer #1 (Remarks to the Author):

This manuscript introduces thermodynamically explainable representations of AI (TERP) as a means to choose simplified models that explain what complex machine learned models have learned. The approach is intended to balance how unfaithful the simplified model is to the full model with how complex the simplified model is using a flexible parameter akin to a temperature. I have an impression there is something to the work, and am thoroughly confused by the current draft.

I wonder if the title and T in TERP should be changed to “thermodynamically-inspired”?

The use of subscripts n and k in the Results (e.g. section A anb B) is quite confusing. It seems that f_k is sometimes used to refer to either 1) a set called f with n parameters, of which k are non-zero or 2) the kth element of a set called f. This creates ambiguities like whether the numerator of the equation for p_k is the absolute value of a single parameter called f_k or the magnitude of a vector called f_k (i.e. with n elements, k of which are non-zero). Likewise, it is unclear if S (equations 4 and 5) is a function of a single model (set called f with k non-zero elements) or a set of models with k ranging from 1 to n.

Confusion mounts in Section C when θ is redefined and then discussed as it relates to the previous definition of θ.

Better intuition for how the characteristic temperature is used to choose k should be given. Its not obvious from the equation or Figure 2 how the difference in characteristic temperatures identifies k where ζ_min is insensitive to temperature. The dependence of the equation on temperature is non-obvious, and the figure gives more information on he dependence of ζ on k than on temperature. Calling on the analogy with thermodynamics wont help for a broad audience, many of whom haven’t thought deeply about thermodynamics recently, if at all.

Figure 1 should show ζ and the characteristic temperature as a function of k so the reader can see how k=3 was chosen for each of the example points A, B, and C.

I’m not sure focusing on transition states in Figure 1 makes sense. It gives the impression that TERP identifies what degrees of freedom distinguish different classifications, when I think its really identifying which degrees of freedom were most important for determining the classification. By definition, its unclear what classification to apply to a transition region. If I’m right here, the choice of focusing on transition states in the first example may contribute to my and others confusion later on.

I don’t understand what Figure 1e is showing. What are “explanation class” and “not explanation class in panel e?

In Figure 3f-g, what is the similarity being measured between? Is the y-value for each trial an average across a set of data or for a single datapoint? Are new noise features being added (e.g. akin to having extra dihedral angles) and then selected out using TERP (e.g. they get zero weight)? Also, its confusing

that the color for pure noise in 3f is the best case scenario if 3g, as I'm naturally drawn to compare lines with the same color between the two plots.

Understanding section E is difficult due to a lack of information. In the first example, it seemed like TERP did a good job of finding which degrees of freedom distinguished classes of data. I'm not sure what the degrees of freedom and classes are in this second example. What are the inputs and outputs of MobileNets? How do labels like 'smiling' relate to the inputs/outputs of MobileNets? Without this information, I'm not really clear how TERP was applied. When you say k=3 was best for smiling, what were the n options from which k was selected? The text and caption don't really help me beyond staring at the figure and trying to guess what the authors intended to do… The result is that I imagine the authors could do some cool things with my data, but I'm not sure they could and I'm very unclear on how I'd try to use their approach myself.

Shouldn't the first author's image be used in figure 4 instead of the last author's? (mostly joking)

I'm equally confused by section F, and the tiny insets are annoying.

In figures 4d, 4e, and 5a how were the temperatures selected?

Reviewer #2 (Remarks to the Author):

==============
= SUMMARY
==============

The manuscript proposes TERP a post-hoc explanation method inspired in Thermodynamics.
One of the main notions of the work is that model complexity (as in number of model parameters) is not necessarily a good proxy for human-interpretability (as in intelligibility). To address this problem, "interpretation entropy" is proposed as key component of the proposed TERP method. In the manuscript it is stated that this entropy term can be used to quantify the degree of human interpretability of any linear model.

In practice, interpretation entropy is balanced against interpretation unfaithfulness. Unfaithfulness is defined based on the correlation of a surrogate model produced via a input-perturbation method and the original model. On the other hand, entropy is defined as the expectation of self-interpretability penalty of all the features considered in the interpretation process.

Experiments conduced on molecular, text and image datasets show the capabilities of the proposed method when applied on different architectures and on different data modalities.

PROs
+ The proposed method is sound

+ Presentation of the manuscript is clear and its content has a good flow.
+ The proposed method is applicable on multiple architectures

CONs
- Positioning w.r.t. existing efforts is limited
- Unsupported claims of human-understandability of the produced explanations

=============
= Concerns
==============

Positioning with respect to existing efforts is almost non-existent. Therefore, besides very specific comments and claims distributed throughout the manuscript based on the content of the manuscript it is hard to position the proposed method in the literature. This this is something critical that must be addressed.

The manuscript does a good job on the validation of the manuscript by testing the capabilities of the proposed methods in datasets with different modalities of data (molecular simulation data, image classification in CelebA, text classification in the AG's news corpus). Unfortunately, the discussion related to each of the results obtained from these experiments is just too shallow as to contribute to a better understanding of the proposed method.

Regarding to the evaluation above, when testing on visual data (Sec. D) the manuscript draws conclusions by only looking at the output produced by three examples(datapoints) at the boundary between three states of the problem at hand. Although interesting, in its current form, the analysis fails to show whether the observation made generalizes to all the datapoints on that region. If possible, I would suggest doing a more comprehensive analysis.

Moreover, explaining the predictions of a given model is a problem that has received significant attention in recent years. At the moment there is no quantitative comparision with respect to related efforts from the literature which raises questions on how better the proposed method really is. Complementing this validation, Adebayo et al., NeurIPS'18, proposed a set of sanity checks to assess whether a given explanation method produces valid explanations. The validation of the proposed method would be strengthened if it passed the proposed method through these sanity checks.

In several parts of the manuscript, there is the claim that the proposed method produces explanations with higher intelligibility (human-understandability / human-interpretability). Following the literature, a claim like this would require a user study to assess the level to which the produced explanations are indeed more intelligible for humans. Unfortunately, no experiment of this type is in place.

In several parts the manuscript states to be addressing the interpretation task (i.e. figuring out what a

model has learned) when in fact it is addressing the explanation task (i.e. justifying the predictions made by a model). This must be corrected prior publication.

In the introduction the manuscript states "Recent approaches that address this issue can be classified into two categories: (a) AI models that are inherently explainable (XAI), or (b) post-hoc interpretation schemes for AI models that are not inherently explainable" Please note that the second group of methods, the post-hoc ones, is the one that is commonly linked to XAI.

Response to reviewer 1

We thank the reviewer for valuable feedback and for appreciating the significance of our work. The reviewer asked very relevant questions and made excellent suggestions which we have considered fully. Below we provide a detailed point-by-point response to the reviewer's comments. All comments are provided in italics followed by our response in bold after an asterisk. Any changes in the manuscript in response to the comments of this reviewer have been indicated using the color blue.

____

This manuscript introduces thermodynamically explainable representations of AI (TERP) as a means to choose simplified models that explain what complex machine learned models have learned. The approach is intended to balance how unfaithful the simplified model is to the full model with how complex the simplified model is using a flexible parameter akin to a temperature. I have an impression there is something to the work, and am thoroughly confused by the current draft.

*** We thank the reviewer for carefully reading our manuscript and appreciate the valuable feedback.**

I wonder if the title and T in TERP should be changed to "thermodynamically-inspired"?

***Thank you for this excellent suggestion. We agree that putting a clarification that our inspiration comes from thermodynamics would be beneficial and should be highlighted in the title and the method name (T in TERP). Thus, we have adopted the term "Thermodynamics-inspired" in our resubmission.**

The use of subscripts n and k in the Results (e.g. section A anb B) is quite confusing. It seems that f_k is sometimes used to refer to either 1) a set called f with n parameters, of which k are non-zero or 2) the kth element of a set called f. This creates ambiguities like whether the numerator of the equation for p_k is the absolute value of a single parameter called f_k or the magnitude of a vector called f_k (i.e. with n elements, k of which are non-zero). Likewise, it is unclear if S (equations 4 and 5) is a function of a single model (set called f with k non-zero elements) or a set of models with k ranging from 1 to n.

***Thank you for this excellent observation and we understand your concerns. To distinguish between the two cases you mentioned, we are now using subscript 'k' to denote the k-th element of an ordered set (e.g, f_k, and p_k for ordered sets f, and p respectively). Additionally, we denote the linear combination of feature coefficients by 'F' instead of 'f' (Eq. 1) to distinguish from the ordered set 'f'. To improve clarity, we are now using the superscript 'j' to indicate that a specific S (or U, ζ) was calculated from a single fitted linear model with n parameters of which j are non-zero i.e, S^j (or U^j, ζ^j). We have added clarifying statements in the Results section (Sec. II).**

Confusion mounts in Section C when θ is redefined and then discussed as it relates to the previous definition of θ.

Better intuition for how the characteristic temperature is used to choose k should be given. Its not obvious from the equation or Figure 2 how the difference in characteristic temperatures identifies k where ζ_min is insensitive to temperature. The dependence of the equation on temperature is non-obvious, and the figure gives more information on he dependence of ζ on k than on temperature. Calling on the analogy with thermodynamics wont help for a broad audience, many of whom haven't thought deeply about thermodynamics recently, if at all.

***We thank the reviewer for pointing out the confusion. We have provided a much more detailed description of characteristic temperature, θ in (Sec II.C). Essentially θ^j is a measure of change in unfaithfulness per unit change in interpretation entropy. We write down the exact equations for calculating θ^j (Eq. 7,8) for a model with j non-zero coefficients. We also present the equation for calculating the final optimal temperature θ^o. Furthermore, we rename ζ as free energy and add appropriate discussions in (Sec. II.C) to clarify the thermodynamic analogy.**

Figure 1 should show ζ and the characteristic temperature as a function of k so the reader can see how k=3 was chosen for each of the example points A, B, and C.

***We thank the reviewer for the constructive suggestion. Since Figure 1 is an illustration, we do believe the reviewer is actually referring to Figure 3 where the A,B,C example points were highlighted. Under this assumption, we have added a plot (Fig. 3f) showing characteristic temperature θ^j as a function of j. In this case, j=2 generates the optimal interpretation as θ^{j+1}-θ^j is minimized at j=2.**

I'm not sure focusing on transition states in Figure 1 makes sense. It gives the impression that TERP identifies what degrees of freedom distinguish different classifications, when I think its really identifying which degrees of freedom were most important for determining the classification. By definition, its unclear what classification to apply to a transition region. If I'm right here, the choice of focusing on transition states in the first example may contribute to my and others confusion later on.

***We thank the reviewer for the pointing out the confusion. Again, we believe this point is referring to Figure 3 and not Figure 1. We agree that TERP is designed to identify which degrees of freedom are most important to a model for predicting a specific class. However, from a physics perspective, which degrees of freedom matter the most near the transition state is an interesting, and practical question that explains what leads to change in the metastable state class. We chose to address this problem to demonstrate the broad applicability of TERP and discuss how the findings align with theoretical studies by Chandler et al. Furthermore, we agree that its not clear what classification to apply at transition region, but the prediction probability is the most sensitive at the transition state. Once a meaningful neighborhood is generated, the perturbed data**

include points away from the transition state and the presence of a broad distribution of probabilities in neighborhood results in highly accurate linear models. Our findings aligning with previous studies also indicate that our approach was able to generate a good representation of local neighborhood. We conducted a comprehensive analysis of 713 configurations and added clarifying statements in the manuscript to alleviate the confusion.

I don't understand what Figure 1e is showing. What are "explanation class" and "not explanation class in panel e?

***We thank the reviewer for the question and believe this comment also refers to Fig. 3e and not Fig. 1e. In TERP, after choosing to explain a specific prediction from a black-box model, a local neighborhood is generated. Ideally, the neighborhoood prediction probabilities should cover a broad distribution without going to the non-linear regime for the most accurate linear model construction. To visualize that a broad distribution of prediction probabilities are present in the neighborhood we used a cutoff of 0.5 to differentiate between the explanation class (>0.5), and not explanation class (<0.5). We have added a clarifying statement in the manuscript.**

In Figure 3f-g, what is the similarity being measured between? Is the y-value for each trial an average across a set of data or for a single datapoint? Are new noise features being added (e.g. akin to having extra dihedral angles) and then selected out using TERP (e.g. they get zero weight)? Also, its confusing that the color for pure noise in 3f is the best case scenario if 3g, as I'm naturally drawn to compare lines with the same color between the two plots.

***We thank the reviewer for the excellent questions regarding similarity error. To ensure the generated neighborhood is indeed local to the original point being explained and not too far away which could possibly include non-linear effects into the model, TERP computes a similarity metric corresponding to each generated datapoint and uses the similarity values as weights during linear model construction as discussed in Sec. IIA. In Fig. 3h-i (Fig 3f-g in the previous submission), we examined if we corrupted a generated neighborhood (by adding different kinds of noise), how much the similarity would get affected. The y-value for each trial is not for a single data point but an average deviation in similarity before and after corruption for the entire neighborhood as discussed in Eq. 8 in detail. For this analysis, we generated different neighborhoods and calculated similarity error when using euclidean vs. LDA based projection as the distance. For this part, we did not run all the TERP steps. Thank you for pointing out the issue with the color. We agree and are now using matching colors between Fig. 3f and 3g (Fig. 3h and 3i in the resubmission).**

Understanding section E is difficult due to a lack of information. In the first example, it seemed like TERP did a good job of finding which degrees of freedom distinguished classes of data. I'm not sure what the degrees of freedom and classes are in this second example. What are the inputs and outputs of MobileNets? How do labels like 'smiling' relate to the inputs/outputs of

MobileNets? Without this information, I’m not really clear how TERP was applied. When you say k=3 was best for smiling, what were the n options from which k was selected? The text and caption don’t really help me beyond staring at the figure and trying to guess what the authors intended to do… The result is that I imagine the authors could do some cool things with my data, but I’m not sure they could and I’m very unclear on how I’d try to use their approach myself.

We thank the reviewer for bringing this to our attention. This is an image classification problem with the CelebA dataset which is a large collection of human facial images with 40 labelled facial attributes e.g, Eyeglasses, Smiling, Male etc. The black-box model takes images as inputs (RGB values) and is trained to predict the probability of these 40 attributes being present in the image. Since image classifiers learn to recognize local features in images, we divide the image into ‘n’ superpixels (collection of pixels). To generate a perturbed image, we randomly pick superpixels and average out the colors within the superpixe. To construct a linear model from these images and corresponding black-box prediction, an one-hot encoded matrix with ‘n’ columns is created with 0 entry if a superpixel was perturbed and 1 otherwise for linear regression. Explanations generated by TERP would be useful to (i) ascertain confidence in a model (e.g, by comparing what we know about ‘Eyeglasses’ and see if the explanation includes Eyeglasses to determine whether we can trust a model or not, (ii) such explanations can be used to identify systematic patterns or biases (e.g, gender, ethnic) in the training data which could be useful to know in various applications. We have added clarifying comments in Sec. IIE and modified this section extensively.

Shouldn’t the first author’s image be used in figure 4 instead of the last author’s? (mostly joking)

We appreciate the comment of the reviewer. Of course any image can be used for this experiment. We are now using the first author’s image.

I’m equally confused by section F, and the tiny insets are annoying.

We apologize for the confusion in Sef. F and have removed the insets. We have added additional description of the inputs and outputs of the model and discuss usefulness of the generated explanations by TERP.

In figures 4d, 4e, and 5a how were the temperatures selected?
***We thank the reviewer for pointing out possible confusion regarding the choice of temperature. We have added a detailed discussion addressing how to choose temperatures in Sec. IIC.**

<u>Response to reviewer 2</u>

We thank the reviewer for valuable feedback and for appreciating the significance of our work. The reviewer asked very relevant questions and made excellent suggestions

which we have considered fully. Below we provide a detailed point-by-point response to the reviewer’s comments. All comments are provided in italics followed by our response in bold after an asterisk. Any changes in the manuscript in response to the comments of this reviewer have been indicated using the color red.

___

==============
= SUMMARY
==============

The manuscript proposes TERP a post-hoc explanation method inspired in Thermodynamics. One of the main notions of the work is that model complexity (as in number of model parameters) is not necessarily a good proxy for human-interpretability (as in intelligibility). To address this problem, "interpretation entropy" is proposed as key component of the proposed TERP method. In the manuscript it is stated that this entropy term can be used to quantify the degree of human interpretability of any linear model.

In practice, interpretation entropy is balanced against interpretation unfaithfulness. Unfaithfulness is defined based on the correlation of a surrogate model produced via a input-perturbation method and the original model. On the other hand, entropy is defined as the expectation of self-interpretability penalty of all the features considered in the interpretation process.

Experiments conduced on molecular, text and image datasets show the capabilities of the proposed method when applied on different architectures and on different data modalities.

* **We thank the reviewer for thoroughly reading our manuscript and providing an accurate summary of our submitted manuscript.**

PROs
+ The proposed method is sound
+ Presentation of the manuscript is clear and its content has a good flow.
+ The proposed method is applicable on multiple architectures
CONs
- Positioning w.r.t. existing efforts is limited
- Unsupported claims of human-understandability of the produced explanations

***We thank the reviewer for the positive comments and valuable feedback.**

=============
= Concerns
==============

Positioning with respect to existing efforts is almost non-existent. Therefore, besides very specific comments and claims distributed throughout the manuscript based on the content of the manuscript it is hard to position the proposed method in the literature. This this is something critical that must be addressed.

***We thank the reviewer for pointing this out. We agree with the reviewer and have added appropriate discussions in introduction to help position our contribution with respect to the many existing efforts in this important area.**

The manuscript does a good job on the validation of the manuscript by testing the capabilities of the proposed methods in datasets with different modalities of data (molecular simulation data, image classification in CelebA, text classification in the AG's news corpus). Unfortunately, the discussion related to each of the results obtained from these experiments is just too shallow as to contribute to a better understanding of the proposed method.

****We thank the reviewer for the constructive remark. For each of the applications we have added further analysis and discussions (e.g, comparison with saliency maps as baseline, validation through sanity checks in Sec. IIE, a comprehensive analysis of different configurations in Sec. IID, significance and usefulness of the TERP generated results) to help improve clarity of our proposed method.***

Regarding to the evaluation above, when testing on visual data (Sec. D) the manuscript draws conclusions by only looking at the output produced by three examples(datapoints) at the boundary between three states of the problem at hand. Although interesting, in its current form, the analysis fails to show whether the observation made generalizes to all the datapoints on that region. If possible, I would suggest doing a more comprehensive analysis.

***We thank the reviewer for this excellent suggestion. We performed a very extensive and comprehensive analysis considering 713 individual predictions for each of which perturbed neighborhoods with 5000 samples were generated and a complete TERP protocol was implemented. This analysis shows the robustness of TERP, as well as the generalizability of the produced capability. Furthermore the results e.g, the contribution of θ dihedral angle near the transition state between is validated by prior theoretical work of Chandler et al.**

Moreover, explaining the predictions of a given model is a problem that has received significant attention in recent years. At the moment there is no quantitative comparision with respect to related efforts from the literature which raises questions on how better the proposed method really is. Complementing this validation, Adebayo et al., NeurIPS'18, proposed a set of sanity checks to assess whether a given explanation method produces valid explanations. The validation of the proposed method would be strengthened if it passed the proposed method through these sanity checks.

***We thank the reviewer for this excellent suggestion. We agree with this point and computed saliency maps as baseline for comparison with our method. Furthermore, we implemented the suggested work from Adebayo et al. to perform both the sanity checks of data randomization and model parameter randomization. From the results it can be seen that TERP explanations are sensitive to both sanity checks and the generated results are not independent of data and the model.**

In several parts of the manuscript, there is the claim that the proposed method produces explanations with higher intelligibility (human-understandability / human-interpretability). Following the literature, a claim like this would require a user study to assess the level to which the produced explanations are indeed more intelligible for humans. Unfortunately, no experiment of this type is in place.

***We thank the reviewer for raising this concern. While we do believe we are working under established paradigms of human cognition, we have added clarifying comments in the manuscript. In existing literature (Ref 19-21 in revised manuscript) it has been shown that the key limitation in human cognition arises from a bottleneck in the ability to process information. By considering model weights as a probability distribution we can calculate information content using Shannon entropy which we use as the human-interpretability penalty term.**

In several parts the manuscript states to be addressing the interpretation task (i.e. figuring out what a model has learned) when in fact it is addressing the explanation task (i.e. justifying the predictions made by a model). This must be corrected prior publication.

***We thank the reviewer for pointing out this mistake. We have corrected this issue throughout the manuscript and highlight our method addresses the black-box model prediction explanation task. However, we emphasize that our approach constructs linear models which are interpretable per construction and since the final model is a close approximation to the black-box model, the learnt interpretation is used as the black-box model explanation. We discuss this in Sec. II of the manuscript.**

In the introduction the manuscript states "Recent approaches that address this issue can be classified into two categories: (a) AI models that are inherently explainable (XAI), or (b) post-hoc interpretation schemes for AI models that are not inherently explainable" Please note that the second group of methods, the post-hoc ones, is the one that is commonly linked to XAI.

***We thank the reviewer for bringing this to our attention. We agree and have corrected the mistake.**

REVIEWER COMMENTS

Reviewer #1 (Remarks to the Author):

I'm satisfied with the edits.

Reviewer #2 (Remarks to the Author):

I thank the authors for addressing my comments in their rebuttal and for the actions taken. After looking at the provided revised version in detail, I have the following concerns:

Work related to explanainability and interpretability of deep models is vast and continuously appearing in the literature. To maximize the exposure of the proposed method, I would still advice having a dedicated related work section where similar efforts are described in detail and the differences/similarities of the proposed method are explicitly indicated. In my opinion, this would help position the proposed method in the vast literature of the subject matter.

In its current form, the content of the paper is rather flat. This hinders different aspects of the conducted research/study and makes difficult to access specific parts of the paper in an efficient manner. The paper would benefit from following a more standard structure presenting related work, proposed method, Evaluation (protocol/results/discussion). Beyond this, further organizing relevant parts into subsections would help improve the presentation of the content. For instance, each of the coducted experiments could be further divided into 1) experimental description (protocol, dataset, metrics), 2) results 3) discussion. For instance, for the case of Sec. E, the conducted sanity checks could be a separate experiment on its own. Given the growth of the paper as part of the revision process, a better organization of its content is needed to ensure its content can be properly accessed.

Similar to the proposed TERP method, there have been several methods in the literature that aim to provide explanation/interpretation capabilities. However, at this moment, there is no empirical comparison with respect to the state of the art. Consequently, it is not possible to assess whether the proposed method is indeed better that what is already available in the literature.

Some variable letters seem to be re-used, which lends itself to confusion. In Sec. A, variable g is used to refer to predictions of the model. Later in Pag. 3, Left column (just before Eq.(3)), the variable is used to refer to ground-truth. F seems to suffer from a similar issue.

Response to reviewer 1

We thank the reviewer for all the constructive comments in the previous round which helped improve our work significantly. We are pleased to hear that the reviewer is satisfied with our edits and desires no further change.

Response to reviewer 2

We thank the reviewer for valuable suggestions and for appreciating our response. The reviewer made few excellent comments which we have considered fully. Below we provide a detailed point-by-point response to the reviewer's comments. All comments are provided in italics followed by our response in bold after an asterisk. Any changes in the manuscript in response to the comments of this reviewer have been indicated using the color blue.

I thank the authors for addressing my comments in their rebuttal and for the actions taken. After looking at the provided revised version in detail, I have the following concerns:

Work related to explanainability and interpretability of deep models is vast and continuously appearing in the literature. To maximize the exposure of the proposed method, I would still advice having a dedicated related work section where similar efforts are described in detail and the differences/similarities of the proposed method are explicitly indicated. In my opinion, this would help position the proposed method in the vast literature of the subject matter.

* **We thank the reviewer for the excellent suggestion. We agree that having a dedicated 'Related Work' section would improve the organization of the paper. To comply with the sectioning requirements of the journal, we included newly added background and related work in the Introduction section (subheadings not allowed in Introduction). To maintain the flow of the content, we first introduced pioneering research in the black-box explanation field and then discussed related work (model-agnostic explanation approaches). For completeness, we referenced several review papers for readers who might be interested in some of the other methods. In addition to highlighting key differences/similarities in Introduction, in Results we added a new subsection "Comparison with advanced methods demonstrate TERP explanations are unique" where we discuss in detail the difference/similarities between TERP and two of the most widely used model-agnostic explanation methods in literature. We have also removed all section numberings as instructed in the guidelines.**

In its current form, the content of the paper is rather flat. This hinders different aspects of the conducted research/study and makes difficult to access specific parts of the paper in an efficient manner. The paper would benefit from following a more standard structure presenting related work, proposed method, Evaluation (protocol/results/discussion). Beyond this, further organizing

relevant parts into subsections would help improve the presentation of the content. For instance, each of the coducted experiments could be further divided into 1) experimental description (protocol, dataset, metrics), 2) results 3) discussion. For instance, for the case of Sec. E, the conducted sanity checks could be a separate experiment on its own. Given the growth of the paper as part of the revision process, a better organization of its content is needed to ensure its content can be properly accessed.

***We again thank the reviewer for the valuable comment. We fully agree that putting some of the experiments under their own subsections would improve the clarity and flow of the paper. Thus, we have created three new subsections in Results for discussing individual experiments, namely: (i) Dimensionality reduction (LDA) significantly improves neighborhood similarity, (ii) Data and model parameter randomization experiments show TERP explanations are sensitive, and (iii) Baseline benchmark against saliency map shows TERP explanations are reliable.**

Similar to the proposed TERP method, there have been several methods in the literature that aim to provide explanation/interpretation capabilities. However, at this moment, there is no empirical comparison with respect to the state of the art. Consequently, it is not possible to assess whether the proposed method is indeed better that what is already available in the literature.

****We thank the reviewer for pointing out this issue with which we agree fully. We address this by adding a fourth new subsection in Results, “Comparison with advanced methods demonstrate TERP explanations are unique” where we perform experiments to compare TERP results with two established model-agnostic approaches i.e, LIME, and SHAP to validate our method and demonstrate TERP’s new contribution to the field.***

Some variable letters seem to be re-used, which lends itself to confusion. In Sec. A, variable g is used to refer to predictions of the model. Later in Pag. 3, Left column (just before Eq.(3)), the variable is used to refer to ground-truth. F seems to suffer from a similar issue.

***We thank the reviewer for reading our manuscript carefully and pointing out the mistakes. We made necessary corrections and now using g to only represent black-box prediction, and F to only represent approximate prediction coming from a linear surrogate model.**

REVIEWERS' COMMENTS

Reviewer #2 (Remarks to the Author):

I thank the authors for the efforts invested in addressing my review.
The provided revision addresses the concerns I initially had with the manuscript
